# Cytoplasmic crowding drives intracellular viscoelastic increase during epithelial maturation

**DOI:** 10.1126/sciadv.aeh7786

**Published:** 2026-11-04

**Authors:** Gregory Arkowitz, Olivier Destrian, Anis Aggoun, Marc-Antoine Fardin, Pascal Berto, Gilles Tessier, René-Marc Mège, Jean-François Berret, Benoit Ladoux

**Affiliations:** ^1^ Université Paris Cité, CNRS, Institut Jacques Monod, F-75013 Paris, France.; ^2^ Sorbonne Université, CNRS, INSERM, Institut de la Vision, 75012 Paris, France.; ^3^ Aix Marseille Université, CNRS, Centrale Marseille, Institut Fresnel, 13013 Marseille, France.; ^4^ Institut Universitaire de France (IUF), 75005 Paris, France.; ^5^ Université Paris Cité, CNRS, Laboratoire Matière et Systèmes Complexes, F-75013 Paris, France.; ^6^ Department of Physics, Friedrich-Alexander Universität Erlangen-Nürnberg, 91058 Erlangen, Germany.; ^7^ Max-Planck-Zentrum für Physik und Medizin and Max Planck Institute for the Science of Light, 91054 Erlangen, Germany.

## Abstract

The cytoplasm is a crowded, active medium whose mechanical properties underpin essential cellular functions, yet how intracellular rheology adapts during tissue maturation remains unclear. Here, we examine the evolution of cytoplasmic viscoelasticity in Madin-Darby canine kidney epithelial monolayers as cell density increases. Using magnetic rotational spectroscopy, an active microrheology technique, we quantify zero-shear viscosity and elastic modulus throughout monolayer maturation. We uncover a substantial enhancement of intracellular viscoelasticity correlated with increasing cell density and a concomitant rise in cellular dry mass concentration. Hyperosmotic perturbations on subconfluent monolayers reproduce this effect, confirming cytoplasmic crowding as a driver of rheological changes. Notably, intracellular viscoelasticity scales linearly with dry mass concentration, suggesting a direct coupling between internal mechanics and cytoplasmic concentration during epithelial maturation. These findings establish a link between tissue-level organization and intracellular mechanics, highlighting cytoplasmic crowding as a regulator of epithelial homeostasis and providing a framework to connect rheology with tissue behavior.

## INTRODUCTION

The cytoplasm is a crowded heterogeneous biological medium (*1*, *2*) in which key physiological cell functions take place, such as intracellular transport (*3*), protein synthesis (*4*), and metabolic processes including glycolysis (*5*). Furthermore, the cytoplasm is essential to signal transduction, as biochemical or mechanical stimuli from the cell’s external environment are relayed throughout the intracellular medium to enable a response, for instance, through gene expression (*3*, *6*). These active intracellular processes require cytoplasmic fluidity and dynamic spatiotemporal remodeling, while maintaining inner cell compartmentalization and structural integrity through cytoplasmic elasticity (*7*, *8*). Understanding how such a complex active behavior can arise from the cytoplasm’s intrinsic mechanical properties therefore remains an open question (*9*–*11*). Moreover, while the cytoplasm has largely been described as a viscoelastic material (*12*–*14*), the precise contribution of different intracellular components (cytoskeleton, organelles, and condensates) to its rheological properties is still under investigation (*15*–*18*).

Ongoing interest to characterize the cytoplasm as an active material has led to the development of numerous microrheology techniques. These methods can be contact-free, such as Brillouin microscopy and optical coherence elastography (*19*, *20*), or rely on probe internalization within cells which, enable passive or active rheology measurements (*21*). In the case of passive microrheology, intracellular probes under Brownian motion or active transport are tracked through time to infer inherent cytoplasmic properties, such as the effective energy (*22*) or intracellular diffusion (*18*, *23*). Conversely, active microrheology methods, like optical or magnetic tweezers, measure the cytoplasmic mechanical response when subjecting internalized probes to an external force (*24*, *25*). Active methods typically allow for a characterization of cytoplasmic viscoelasticity by measuring the storage and loss moduli within a wide range of frequencies (*11*, *26*).

Along with theoretical simulations, these experimental approaches have offered crucial insight on how intracellular mechanics affect various biological functions. The cytoplasm’s intrinsic viscoelastic nature was shown to play a major role during mitosis, as it facilitates chromosome segregation (*27*) and affects spindle positioning during sea urchin embryo development (*28*). Intracellular viscoelasticity was also reported to be a driving force in the genesis of biomolecular condensates (*29*, *30*) and cytoplasmic subcompartments, for example, in *Neurospora crassa* (*31*). Additionally, these properties affect microtubule turnover (*32*) and can potentially serve as relevant mechanical markers for metastatic cancer cells (*33*). Although such studies have established clear evidence regarding the need for an intracellular mechanical description at the single cell level, there is currently very little knowledge on how intracellular rheology is linked to tissue scale processes.

During epithelial tissue maturation, increases in cell density modulate collective migration (*34*–*36*), traction force generation on the substrate (*37*, *38*), and cell mechanics (*39*, *40*) and drive morphological transitions that establish apico-basal polarity and mature cell-cell junctions (*41*). High-density maturation is also accompanied by a reduction in cell proliferation and motility, termed contact inhibition of proliferation and locomotion, thereby contributing to tissue-scale homeostasis (*42*, *43*). These dynamic collective behaviors occurring during tissue maturation allow epithelia to maintain their barrier function, for example, in the context of wound healing (*44*, *45*). While several studies have linked cell mechanics, probed externally, to epithelial tissue maturation (*39*, *46*), there is currently little information on how this process is affected by intracellular mechanical properties.

In this work, we investigate how the cytoplasmic viscoelastic properties of Madin-Darby canine kidney (MDCK) cells forming an epithelial monolayer evolve as the tissue becomes denser during its maturation. The cytoplasm’s rheology is characterized by performing live measurements of its zero-shear viscosity and elastic modulus through the use of a method based on magnetic rotational spectroscopy (MRS) (*47*–*49*). This active method is particularly well adapted to studying intracellular rheology in tissues, as it enables live measurements of cytoplasmic viscoelasticity simultaneously within multiple cells. We apply this approach to decipher the correlation between inner cell viscoelasticity and cell density within a progressively maturing epithelial monolayer. We also investigate changes in cell volume and dry mass in increasingly dense monolayers, to establish whether the regulation of intracellular rheology during tissue maturation is dependent on cytoplasmic dry mass concentration. Moreover, osmotic perturbations allow us to further quantify the impact of intracellular concentration on cytoplasmic rheology. Together, this work aims at establishing a clear link between cell density and cytoplasmic viscoelasticity, which can be tuned by changes in the intracellular concentration occurring during tissue maturation.

## RESULTS

### MRS method to probe mechanical properties of epithelial monolayer tissues

The active microrheology method known as MRS has been previously applied to characterize cytoplasmic viscoelasticity in single cells (*47*). Consistently with past studies (*33*, *47*), the Maxwell viscoelastic fluid model was used to approximate the cytoplasm’s mechanical behavior, allowing us to determine two rheological parameters: the zero-shear viscosity η_0_ at low frequency (1 to 100 mHz) and the elastic modulus *G*_0_ at high frequency (10 Hz) (Fig. 1A). These measurements are obtained by subjecting internalized micrometric superparamagnetic microwires to a uniform magnetic field at a constant magnitude, continuously rotating around the biological sample at a given frequency (fig. S1). The determination of rheological parameters is enabled by probing a wide range of rotational frequencies to uncover two characteristic dynamic behaviors of internalized microwires (Materials and Methods). In the permanent regime, internalized microwires have a characteristic dynamic transition, as the rotating magnetic field frequency increases. Microwires transition from a synchronous regime at low frequency (movie S1) to an asynchronous oscillatory regime at high frequency (movie S2), as illustrated by the representative angle traces shown in fig. S2. The zero-shear viscosity η_0_ and elastic modulus *G*_0_ are then respectively determined from the measured critical frequency ω_C_ separating both regimes, as well as the microwire’s oscillation amplitude θ_0_ at high frequency (Materials and Methods). Here, MRS was applied to quantify cytoplasmic viscoelasticity across MDCK monolayers spanning a continuum of cell densities and maturation states, in two complementary contexts: proliferative subconfluent monolayers that evolve into dense, confluent tissues, and collectively migrating monolayers exhibiting cell density gradients generated by model wound healing assays (Fig. 1B) (*50*, *51*).

**Fig. 1.**
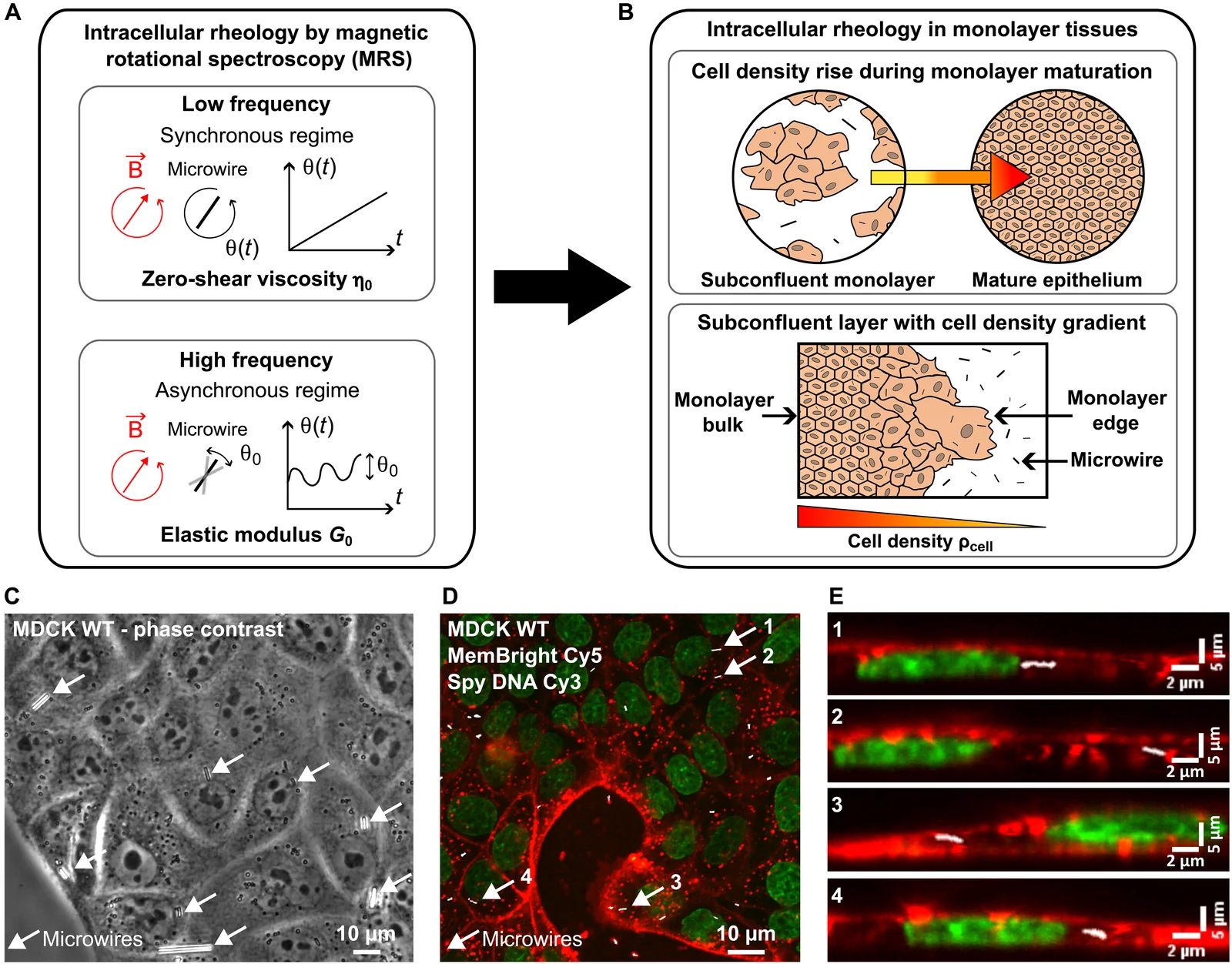
MRS in epithelial monolayer tissues. (**A**) MRS enables the probing of intracellular zero-shear viscosity η_0_ (top) and elastic modulus *G*_0_ (bottom), at respectively low (mHz) and high frequency (10 Hz). At a given critical frequency ω_C_, microwires transition from a synchronous regime to an asynchronous regime, characterized by oscillatory motion. (**B**) MRS is here applied to measure cytoplasmic rheology in the context of MDCK epithelial tissue maturation. Two systems relevant to epithelial maturation were studied to link cytoplasmic rheology to modulations in cell density: a subconfluent monolayer transitioning to a confluent mature epithelium (top) and an initially confined confluent monolayer undergoing unjamming (bottom). (**C**) Subconfluent MDCK monolayer with internalized microwires (marked by white arrows). Scale bar, 10 μm. (**D**) Subconfluent MDCK monolayer with MemBright Cy5 staining (red) and Spy DNA Cy3 (green). Internalized microwires are visualized by thresholding the phase contrast image (white). Scale bar, 10 μm. (**E**) Side view of membrane stained MDCK cells (red) with their nucleus (green) and internalized microwires (white). Numbers at the top left correspond to the microwires indicated in (D). Signals for cell membrane and nucleus are obtained from the average z-projection intensities of the resliced images. Microwire shapes are obtained from the minimum z-projection intensity of the resliced images. Scale bars, 5 μm (vertical) and 2 μm (horizontal).

Superparamagnetic microwires were synthesized by electrostatic complexation of maghemite particles, as previously described (*52*) (Materials and Methods). Microwires had a mean length and diameter of, respectively, *L* = 3.71 ± 0.04 μm and *D* = 0.76 ± 0.01 μm (unless stated otherwise, data reported in the text correspond to the mean ± standard error to the mean, whereas figures show the mean ± standard deviation) (fig. S3, A and B). The magnetic susceptibility parameter Δχ of synthesized microwires was assessed in a water-glycerol solution of known viscosity (fig. S3C) and was used to compute cytoplasmic viscosity and elasticity according to equations reported in Materials and Methods. Microwires were internalized in MDCK monolayers by overnight incubation, with no apparent cell death or change of cell morphology (Fig. 1C). Proper probe internalization was ensured by performing live *z*-stack imaging of MDCK wild-type (WT) cells with live membrane staining (Fig. 1D). Sideview visualization by *z*-stack reslicing (Fig. 1E) revealed an average internalization rate of 85% after overnight incubation. Cells internalized, on average, one to two microwires, although large cells at the monolayer’s edge were sometimes capable of internalizing substantially more probes. Prior transmission electron microscopy images of internalized microwires in NIH/3T3 and HeLa cells indicate that these probes are freely diffusing in the cytosol and are not enclosed in internal membrane compartments (*47*).

### Sharp intracellular viscoelastic increase of MDCK cells in highly dense monolayers

MRS was therefore applied to determine the intracellular viscoelastic properties of MDCK monolayers undergoing maturation. After cell seeding, subconfluent MDCK monolayers progressively spread across the substrate through proliferation and collective migration until reaching full confluence (Fig. 2A). Cell density continues to increase, and thus, cell area gradually decreases as confluent tissues mature toward a homeostatic plateau (Fig. 2A). Quantitatively, this process is reflected by a steady reduction in mean cell area, from *A*_cell_ = 468 ± 26 μm^2^ in subconfluent monolayers to *A*_cell_ = 250 ± 4 μm^2^ at early confluence, followed by stabilization at *A*_cell_ ∼ 167 ± 2 μm^2^ within 12 hours after confluence (Fig. 2B), consistent with previous reports (*53*). Monolayer maturation is also accompanied by a jamming transition where the average cell velocity |*v*|, measured by particle imaging velocimetry (PIV), sharply decreases when reaching confluence (Fig. 2C). This jamming transition can be visualized by plotting the mean squared displacement (MSD) of individual cells through time (fig. S4A). MDCK cells, which have a super-diffusive directed motion (α = 1.6) in a subconfluent state, transition to a caged subdiffusive motion ∼4 hours postconfluence (α = 0.9) (fig. S4A). This jamming transition dynamic was unaltered in MDCK monolayers with internalized microwires (fig. S4B). Following monolayer jamming, epithelial maturation is further characterized by decreased cell proliferation, as previously reported (*53*). The proportion of cells entering a quiescent G0 state increases during monolayer maturation (31 ± 1% of G1/G0 cells in subconfluent tissues compared to 56 ± 2% in dense monolayers, 20 hours postconfluence) (Fig. 2D). This contact inhibition of proliferation at high cell density is further reflected by a significant decrease of cells in S phase (Fig. 2D). Overall, these changes in cell proliferation, size, and migration led us to distinguish three stages of monolayer maturation for microrheology experiments: subconfluent layers (up to 10 hours preconfluence), monolayers at onset of confluence (2 hours preconfluence to 2 hours postconfluence), and densely confluent monolayers (over 12 hours postconfluence).

**Fig. 2.**
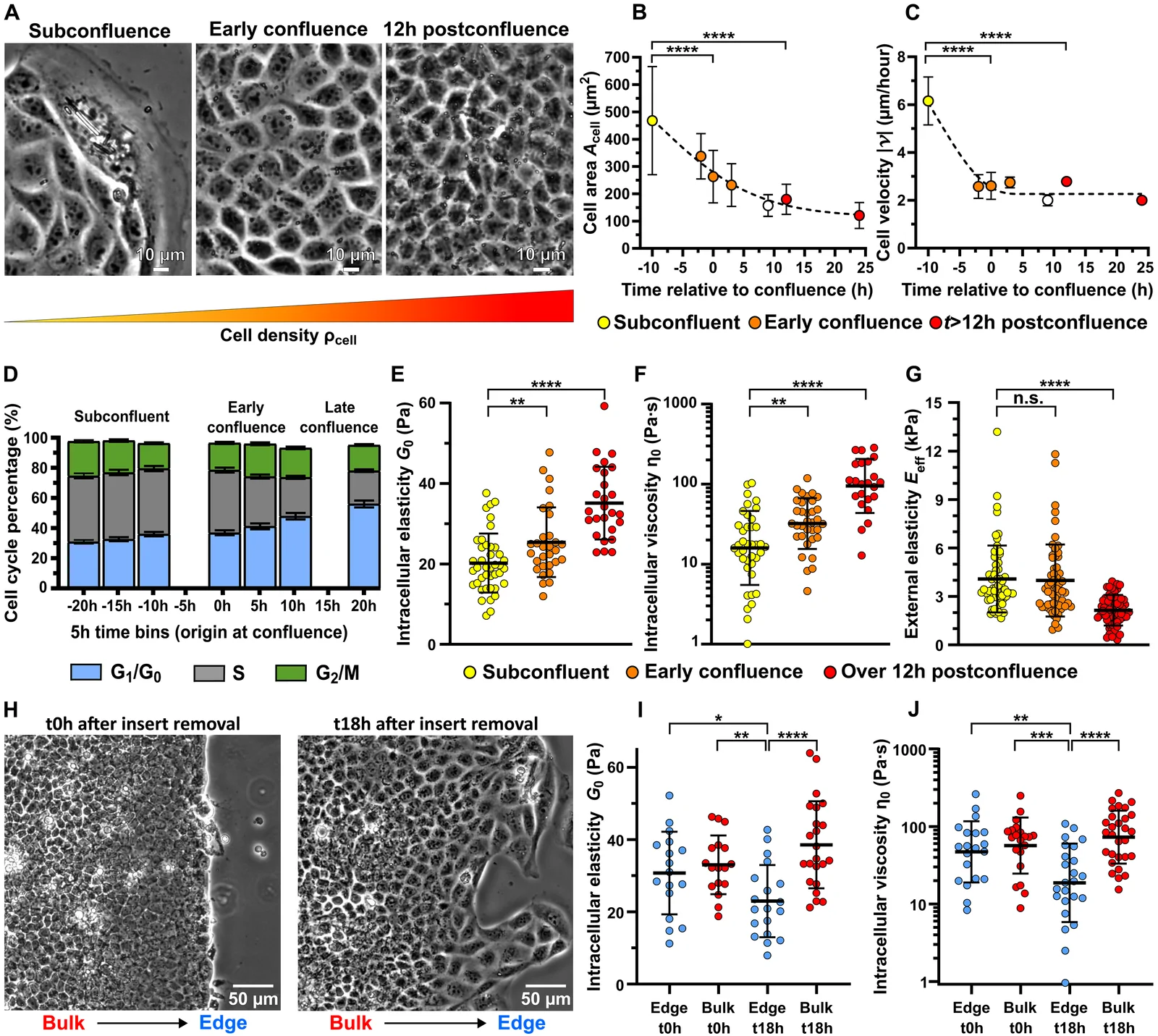
Sharp intracellular viscoelastic strengthening of MDCK cells in highly dense monolayers. (**A**) MDCK monolayer at different stages of confluence and cell density. From left to right: Monolayer at subconfluence (10 hours preconfluence), monolayer at onset of confluence, and monolayer in late stages of confluence (12 hours postconfluence). Scale bar, 10 μm. (**B**) Cell area during different stages of confluence (*N* ≥ 3 per confluence category). Experimental points are fitted with an exponential decay function (dotted line), and white data points correspond to the intermediate condition between early and late confluence (between 2 and 12 hours postconfluence). (**C**) Average cell velocity (PIV) during different stages of confluence (*N* ≥ 2 per confluence category). Experimental points are fitted with a spline function (dotted line). (**D**) Cell cycle distribution (FACS) as a function of the monolayer’s relative time to confluence (*N* = 10). (**E**) Intracellular elasticity (*n* ≥ 26, *N* = 3 for each condition) and (**F**) intracellular viscosity (*n* ≥ 23, *N* = 3 for each condition) measured by MRS for different stages of confluence. (**G**) External elasticity measured by nanoindentation for different stages of confluence (*n* = 58, *N* = 2). (**H**) Phase contrast images of an MDCK monolayer during a wound healing experiment: initially after confinement release (left) and 18 hours postconfinement removal (right). Scale bars, 50 μm. (**I**) Intracellular elasticity (*n* ≥ 17, *N* = 3 for each condition) and (**J**) intracellular viscosity (*n* ≥ 20, *N* = 3 for each condition) at the monolayer’s edge (blue) and bulk (red), immediately after insert removal and 18 hours postremoval. Data points for all panels are shown with their means and SD, at the exception of (F) and (J), which show the geometric means and GSD. Unpaired Welch’s *t* test for (B), (C), and (I). Unpaired Student’s *t* test for (E). Unpaired Welch’s *t* test on log values for (F) and (J) (log-normal distribution). Mann-Whitney test for (G). ****P < 0.0001; ***P < 0.001; **P < 0.01; *P < 0.05; not significant (n.s.), *P* ≥ 0.05. h, hours.

The mechanical properties of MDCK cells in monolayers were probed internally by MRS and externally by nanoindentation (Materials and Methods). Results obtained by MRS point to a significant increase in cytoplasmic viscosity and elasticity in highly dense monolayers (henceforth referred to as an increase of “cytoplasmic viscoelasticity”). Intracellular elasticity rises from *G*_0_ = 20 ± 1 Pa in subconfluent layers to *G*_0_ = 35 ± 2 Pa in mature monolayers, while intracellular zero-shear viscosity jumps from η_0_ = 16 ± 3 Pa·s to η_0_ = 95 ± 19 Pa·s (Fig. 2, E and F). This significant increase in both rheological parameters for highly dense monolayers (red) appears to take place subsequently to the jamming transition occurring during confluence onset (orange), as determined by PIV and MSD (Fig. 2, C, E, and F, and fig. S4A). Moreover, this shift in intracellular mechanical state coincides with the inhibition of cell proliferation, characteristic of epithelial monolayer maturation, starting ∼10 hours postconfluence (Fig. 2, D to F). Notably, cells in highly dense monolayers had a significantly softer response to indentation compared to cells in subconfluent or barely confluent monolayers, shown by a decrease in external effective Young’s modulus from *E*_eff_ = 4.1 ± 0.3 kPa to *E*_eff_ = 2.1 ± 0.1 kPa (Fig. 2G). This result is consistent with previous studies (*39*) and highlights the lack of direct correlation between changes in external cell elasticity and the cytoplasm’s mechanical properties during MDCK monolayer maturation.

The substantial increase of cytoplasmic viscoelasticity following monolayer jamming (Fig. 2, C, E, and F) led us to hypothesize that this intracellular strengthening during monolayer maturation is closely coupled to changes in cell area, rather than by the layer’s transition from subconfluence to confluence. To test this hypothesis, MRS measurements were then performed on monolayers with a cell size gradient, imposed by the initial confinement and subsequent removal of two-well culture inserts (Materials and Methods), mimicking the closure of a rectangular wound gap. More specifically, intracellular rheology was characterized at the monolayer’s edge and bulk, respectively below and above 200 μm from the migrating edge (fig. S5A), upon initial insert removal (t0h) and 18 hours postremoval (Fig. 2H). The distinctiveness of these two monolayer regions was confirmed by quantifying the recovery of cell motility and effective cell spreading at the edge, in contrast to the bulk (fig. S5, B and C). Highly compacted cells (*A*_cell_ = 166 ± 3 μm^2^), upon initial confinement release (t0h), displayed a significantly higher intracellular elasticity (*G*_0_ = 31 ± 3 Pa) and viscosity (η_0_ = 47 ± 9 Pa·s) compared to more spread out cells (*A*_cell_ = 455 ± 15 μm^2^) at the migrating edge, 18 hours post–insert removal (*G*_0_ = 23 ± 2 Pa for elasticity, η_0_ = 19 ± 6 Pa·s for viscosity) (Fig. 2, I and J). This result reveals the reversible nature of intracellular viscoelastic strengthening during dense tissue spreading and further confirms the interplay of cytoplasmic viscoelasticity with cell area. Furthermore, cytoplasm viscoelasticity at the monolayer’s edge during its migration (t18h) is significantly lower than in its bulk (*G*_0_ = 23 ± 2 Pa compared to *G*_0_ = 39 ± 3 Pa for elasticity, η_0_ = 19 ± 6 Pa·s compared to η_0_ = 73 ± 15 Pa·s for viscosity). These findings demonstrate a direct coupling between intracellular rheology, cell size, and tissue dynamics.

To identify the mechanisms underlying cell size–dependent changes on intracellular rheology, we examined the contribution of major cytoplasmic features known to drive cell mechanical properties. The cytoskeleton, including actin filaments and microtubules, forms a load-bearing network that governs intracellular elasticity, force transmission, and cytoplasmic organization (*17*, *54*–*56*). Therefore, we chose to quantify the viscoelastic impact of these structures using targeted cytoskeletal-perturbing drugs (4 μM cytochalasin D or latrunculin A for actin disruption and 33 μM nocodazole for microtubule depolymerization) (fig. S6A). Consistent with the established role of the actin cortex in cell mechanics (*57*, *58*), nanoindentation measurements revealed a marked decrease in external cell elasticity upon actin disruption (4 μM cytochalasin D) in subconfluent monolayers, whereas the already low external elasticity remained largely unaffected in highly dense monolayers (fig. S6B). This suggests that although decreased external elasticity during monolayer maturation (from ∼4 to ∼2 kPa) is likely linked to actin remodeling, residual stiffness at high cell density may reflect contributions from membrane tension (*59*) and altered mechanical coupling mediated by cell-cell adhesions (*60*). Notably, however, intracellular viscoelasticity measured by MRS was largely insensitive to cytoskeletal perturbations in subconfluent monolayers, at the exception of microtubule depolymerization with nocodazole, which decreased intracellular viscosity from η_0_ = 16 ± 3 Pa·s to η_0_ = 9 ± 2 Pa·s (fig. S6, C to G). In parallel with cytoskeletal perturbations, we assessed the effect of polysome disruption on intracellular viscoelasticity, given their polymer-like structure and their speculated substantial contribution to cytoplasmic macromolecular crowding due to their size and abundance (*23*, *61*). Polysome dissociation with puromycin (184 μM) in subconfluent monolayers produced a marked reduction in intracellular elasticity from *G*_0_ = 20 ± 1 Pa to *G*_0_ = 16 ± 1 Pa (fig. S6H). Additionally, this effect was even more pronounced when perturbing polysomes in highly dense confluent monolayers, as the intracellular elasticity dropped from *G*_0_ = 38 ± 2 Pa to *G*_0_ = 27 ± 2 Pa. Overall, these results reveal a substantial contribution of macromolecular crowding to cytoplasmic elasticity (i.e., high elastic modulus) at different stages of monolayer maturation. Hence, we next investigated the evolution of physical parameters relevant to cytoplasmic crowding, such as cell volume and dry mass, as we hypothesized that an increase in intracellular viscoelasticity during monolayer maturation could be driven by changes in cytoplasmic concentration.

### Cell volume and dry mass decrease in progressively maturing MDCK monolayers

Having found that major intracellular crowders, like polysomes, affect intracellular rheology in low– and high–cell density monolayers, we therefore asked whether density-dependent rigidification during epithelial maturation could be associated to changes in overall intracellular concentration during cell size reduction. To test this hypothesis, we quantified parameters directly allowing to infer intracellular concentration: cell volume and dry mass. Dry biomass in MDCK monolayers was monitored by quantitative phase imaging (QPI) (Materials and Methods). To enable reliable phase-background subtraction, including in fully confluent samples, MDCK monolayers were grown on fibronectin-patterned glass coverslips (Fig. 3A). Cell spreading areas in samples used for QPI quantification were comparable to those observed in MRS experiments, with a significant decrease in cell size as density increases (Figs. 3B and 2B). During monolayer maturation, we noticed increased cell-cell heterogeneity in QPI signal across the monolayer, likely due to cell extrusion events at elevated cell density (*62*, *63*), which locally alter projected mass distribution for this two-dimensional measurement method (Fig. 3A; Materials and Methods). Nevertheless, QPI results reveal a significant drop in mean cellular dry mass during cell density increase, going on average from *m*_cell_ = 421 ± 10 pg in low-density subconfluent monolayers, to *m*_cell_ = 332 ± 11 pg at the onset of confluence and *m*_cell_ = 296 ± 8 pg in high-density confluent monolayers (Fig. 3C). This corresponds, on average, to a cell dry mass loss of 21 ± 3% and 30 ± 3%, respectively, in low– and high–density confluent monolayers compared to subconfluent patterns.

**Fig. 3.**
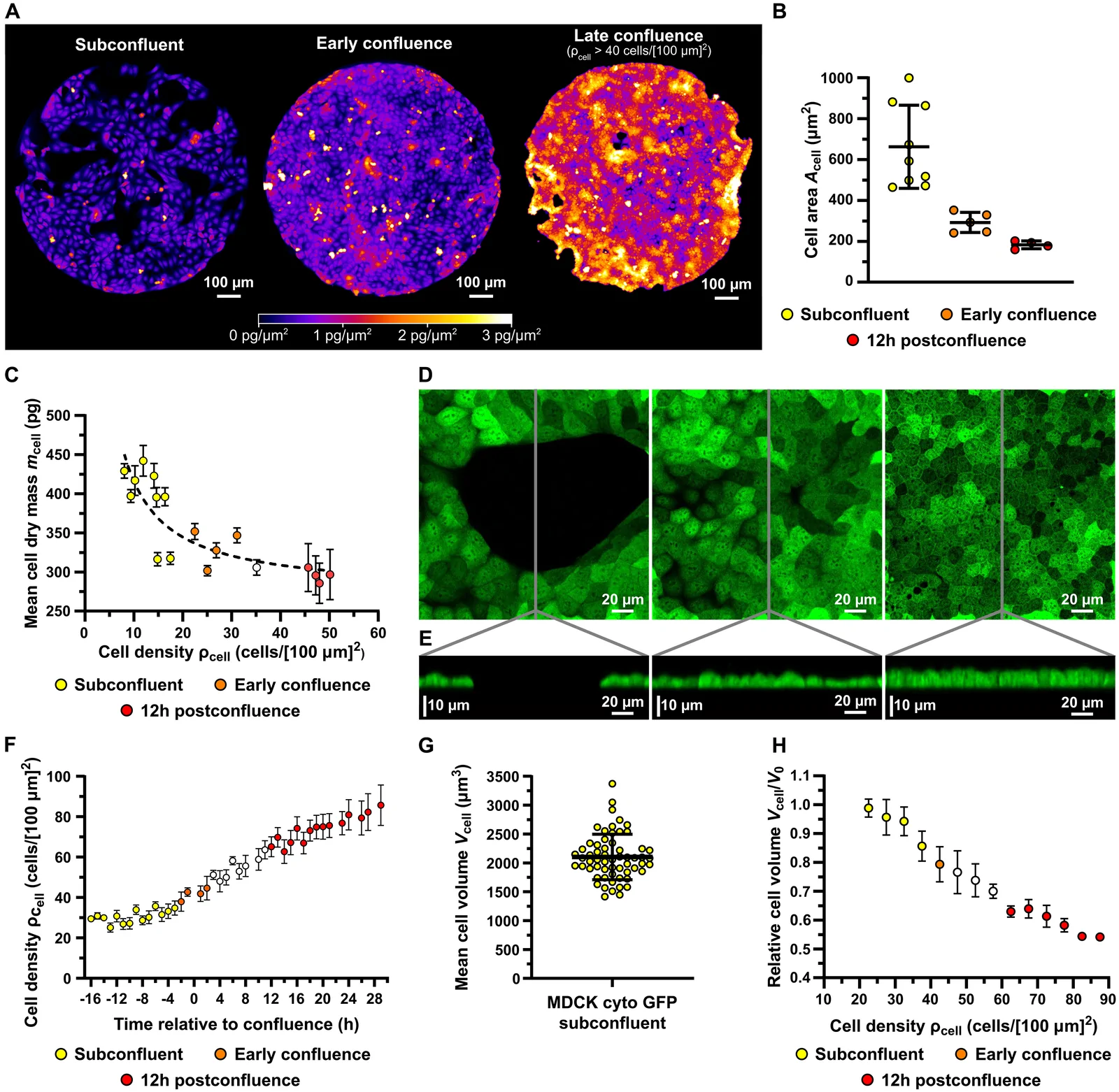
Cell volume and dry mass decrease in progressively maturing MDCK monolayers. (**A**) QPI of MDCK cells grown at different densities on disk-shaped fibronectin patterns printed on glass coverslips. From left to right: Subconfluent monolayer, confluent monolayer at low density, confluent monolayer at high density (above 40 cells per [100 μm]^2^). Color code corresponds to the measured dry mass surface density in picograms per square micrometer. Scale bars, 100 μm. (**B**) Mean cell area quantified by CellPose of MDCK cells grown on circular glass fibronectin patterns [*N* ≥ 4 per sample type shown in (A)]. (**C**) Mean cell dry mass as a function of cell density (*N* ≥ 4 per sample type). Experimental points are plotted with SD and are fitted with a linear rational function with two adjustable parameters [*f*(*x*) = *a*/*x* + *b*, dotted lines]. (**D**) MDCK with cytosolic free GFP at different stages of confluence. From left to right: Monolayer at subconfluence (10 hours preconfluence), monolayer at onset of confluence, and monolayer in late stages of confluence (over 12 hours postconfluence). Scale bars, 20 μm. (**E**) Side view of the corresponding images from (D) obtained from averaging the *z*-projection intensities of resliced images. Horizontal scale bars, 20 μm; vertical scale bars, 10 μm. (**F**) Cell density evolution of MDCK cytosolic free GFP across different stages of confluence (*n* = 10, *N* = 2). (**G**) Absolute mean cell volume of MDCK cytosolic free GFP cells in subconfluent monolayers (*n* = 60, *N* = 4). (**H**) Relative cell volume, normalized by the average initial subconfluent cell volume, as a function of cell density (*n* = 10, *N* = 2). White data points in (C), (F), and (H) correspond to the intermediate condition between early and late confluence (between 2 and 12 hours postconfluence). All represented data points correspond to mean values with SDs.

To determine whether this decrease in dry mass was solely due to a decrease in cell size or was accompanied by a change in cellular dry mass concentration, we quantified cell volumes. Three-dimensional time-lapse imaging was performed on monolayers of MDCK cells expressing cytosolic green fluorescent protein (GFP), as they progressed from a subconfluent to a highly dense tissue (from left to right, Fig. 3D). Consistently with previous studies (*53*), we observed a marked increase of cell height concomitantly with a decrease in projected cell area, as monolayer cell density rose through time during maturation (Fig. 3, D to F). The acquired three-dimensional time-lapse datasets were analyzed using a custom thresholding algorithm to extract total monolayer volume, which was normalized by cell number to obtain mean cellular volume (Materials and Methods) (*18*). Owing to substantial field-to-field variability (Fig. 3G), relative cell volume was calculated by normalizing to each field’s initial subconfluent value (Fig. 3H). During maturation, MDCK cells exhibited a significant volume reduction, decreasing, on average, by 21 ± 3% at early confluence and 41 ± 2% over 12 hours postconfluence (Fig. 3H). Combining these measurements with the mean absolute subconfluent cell volume (Fig. 3, G and H) yielded time-resolved estimates of cellular volume (fig. S7A) and revealed a concurrent doubling of mean cell height as tissues densified (fig. S7B). As a result, cells in highly dense monolayers contained less dry biomass yet underwent an even greater reduction in volume, resulting in a significant increase in intracellular dry mass concentration. This concentration shift provides a plausible physical explanation for the rise in cytoplasmic viscoelasticity at high cell density, as the rheology of heterogeneous aqueous suspensions like the cytoplasm is highly dependent on concentrations of crowding elements, which can drive an effective liquid-glass transition (*64*, *65*).

### Cell volume modulation through hypertonic stress leads to high intracellular viscoelasticity

To confirm the role of intracellular concentration on changes in cytoplasmic viscoelasticity, we experimentally manipulated cell volume by subjecting MDCK monolayers to various hypertonic stress conditions. Hypertonic shocks lead to a sharp decrease of cell volume by water efflux without affecting cell dry mass (*66*). Subconfluent islands of MDCK cells were submitted to hypertonic shocks (from 0.35 to 1 osmol/liter), by adding sucrose to the isotonic culture medium (0.3 osmol/liter). As illustrated for a 0.6 osmol/liter medium switch, cells flattened while retaining most of their basal area, indicating volume loss (Fig. 4, A and B). Quantitatively, subconfluent cells subjected to hypertonic shocks of increasing intensity displayed a significant volume loss *V*_cell_ relative to their isotonic state *V*_0_, down to 49 ± 4% of their initial volume for 1 osmol/liter shocks (Fig. 4C). The relative volume plotted as a function of osmotic stress π = *P*_hyper_/*P*_iso_, with *P*_hyper_ and *P*_iso_ as the osmotic pressure under hypertonic and isotonic conditions, was fitted with Ponder’s relation $\frac{v_{\text{hyper}}}{v_{\text{iso}}}=\frac{R}{\pi }+\left(1-R\right)$, with *R* = 0.7 as the ratio of osmotically active volume to total cell volume (*67*).

**Fig. 4.**
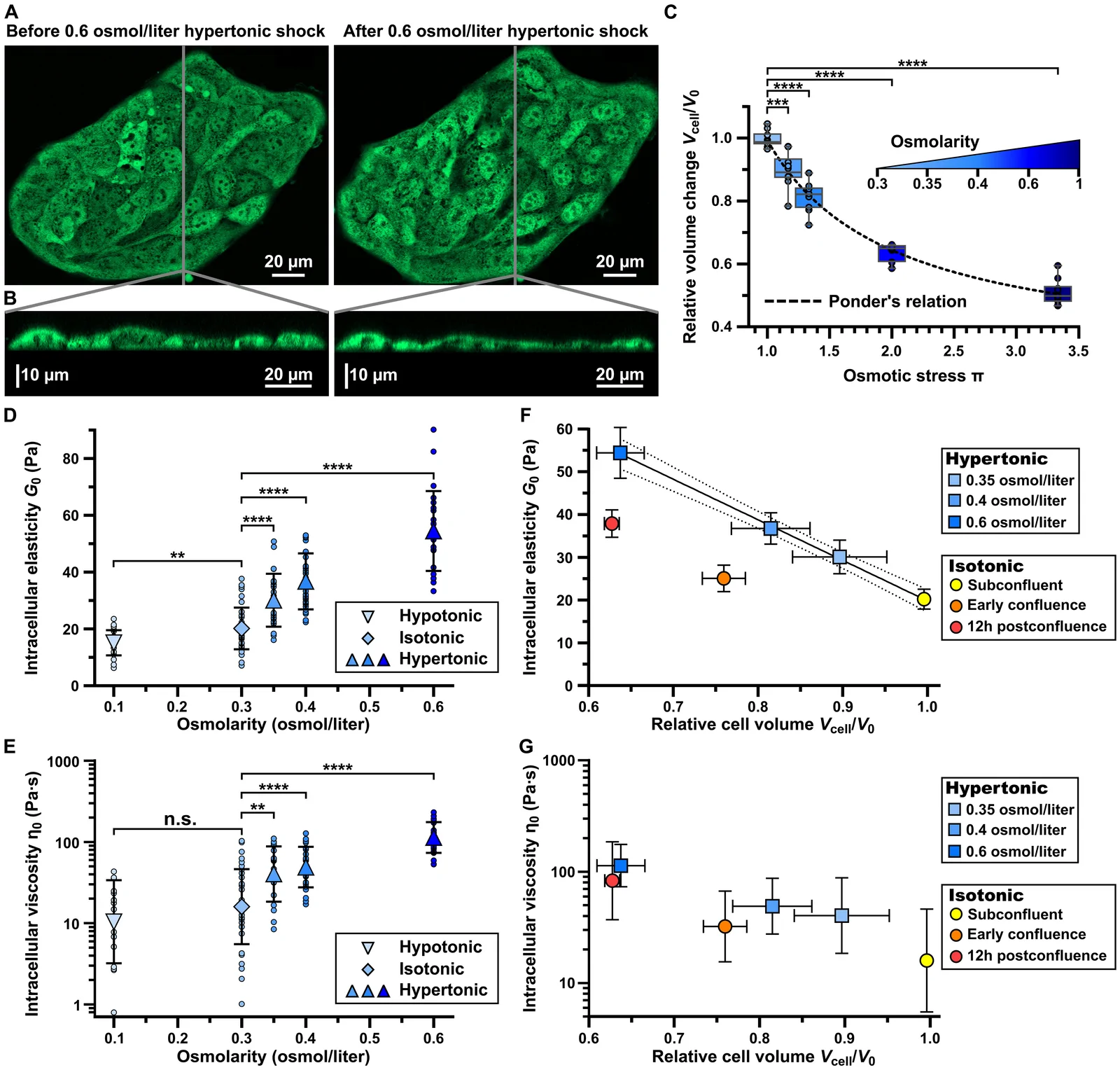
Cell volume modulation through hypertonic stress leads to high intracellular viscoelasticity. (**A**) Subconfluent monolayer of MDCK cytosolic free GFP in 0.3 osmol/liter isotonic medium (left) and 0.6 osmol/liter hypertonic medium (right). Scale bars, 20 μm. (**B**) Side view for the corresponding images from (A), obtained from averaging the *z*-projection intensities of resliced images. Horizontal scale bars, 20 μm; vertical scale bars, 10 μm. (**C**) Relative cell volume change in MDCK subconfluent monolayers subjected to hypertonic shocks of different intensities (*n* = 10, *N* = 2 for each type of hypertonic perturbation). Experimental points are shown with box plots centered around the mean value and are fitted with Ponder’s relation, with *R* = 0.7 as the ratio of osmotically active volume to total cell volume. (**D**) Intracellular elasticity (*n* ≥ 21, *N* ≥ 2 for each condition) and (**E**) intracellular viscosity (*n* ≥ 16, *N* ≥ 2 for each condition) of MDCK cells forming subconfluent monolayers subjected to 0.1 osmol/liter hypoosmotic shock or 0.35, 0.4, 0.6 osmol/liter hyperosmotic shocks. (**F**) Intracellular elasticity and (**G**) intracellular viscosity plotted against the cell volume relative to isotonic subconfluent conditions, for hypertonic shocks (shades of blue) or isotonic monolayers at various stages of confluence (yellow to red). Intracellular elasticity for hypertonic conditions in (F) was fitted with a linear regression with 95% confidence interval. Data points for intracellular elasticity (D) and intracellular viscosity (E) are respectively represented with their means with SD and geometric means with GSD. Data points for (F) and (G) correspond respectively to the means and geometric means with 95% confidence intervals. One-sample *t* test with a null hypothesis value of 1 for (C). Unpaired Welch’s *t* test on intracellular elasticity values (Gaussian distributions) in (D) and on log values of intracellular viscosity (log-normal distributions) in (E). ****P < 0.0001; ***P < 0.001; **P < 0.01; n.s., *P* ≥ 0.05.

MRS measurements were performed for each of these aforementioned hypertonic conditions, along with a hypotonic shock at 0.1 osmol/liter, carried out by diluting isotonic medium with MilliQ deionized water (Fig. 4, D and E). The hypotonic perturbation substantially lowered intracellular elasticity from *G*_0_ = 20 ± 1 Pa to *G*_0_ = 15 ± 1 Pa, but did not significantly affect intracellular viscosity (Fig. 4, D and E). Cells subjected to hypotonic shocks are known to recover faster than under hypertonic conditions, which could explain the limited impact on intracellular viscosity (*68*). Hence, we focused on MRS experiments performed under hypertonic conditions. Cells subjected to a 1 osmol/liter hypertonic shock instantly immobilized microwires (yellow arrows), indicating complete cytoplasmic jamming (movie S3). Conversely, reintroducing isotonic medium enabled the visual recovery of microwire motion within minutes, as initial cell volume was restored (movie S4). Lower intensity hypertonic shocks (0.35, 0.4, and 0.6 osmol/liter) hindered microwire motion while still allowing for quantitative measurements of intracellular viscoelasticity (Fig. 4, D and E). Hypertonic stress of increasing intensity resulted in a consistent rise in intracellular elasticity and zero-shear viscosity, with values soaring respectively to *G*_0_ = 54.4 ± 2.9 Pa and η_0_ = 114 ± 10 Pa·s after a 0.6 osmol/liter shock (compared to *G*_0_ = 20 ± 1.1 Pa and η_0_ = 16 ± 3 Pa·s for isotonic conditions). The ability to strongly modulate intracellular rheology by altering cell volume therefore confirms the pivotal role of intracellular concentration on cytoplasmic viscoelasticity in monolayer tissues.

Both intracellular elasticity and viscosity were then plotted against changes in cell volume for isotonic monolayers at various densities (long evolution) and subconfluent samples subjected to hypertonic stress (short evolution) (Fig. 4, F and G). Under both conditions, cell shrinkage was strongly associated with increased intracellular viscoelasticity. During hypertonic shocks, intracellular elasticity scaled linearly with volume loss (*R*^2^ = 0.99; Fig. 4F). In contrast, maturing monolayers exhibited a weaker, nonsuperimposable relationship regarding the dependence of intracellular viscoelasticity with cell volume loss (Fig. 4, F and G). These differences indicate that respective contributions of cell volume, dry mass, and intracellular concentration are not equivalent in both situations. Under hypertonic stress, dry mass is largely conserved while volume decreases, leading to a rise in intracellular dry mass concentration. During monolayer maturation, however, intracellular dry mass concentration still increases, but through a combined effect of volume loss and partial mass reduction (Fig. 3, C and H). This suggests that dry mass concentration is the more relevant parameter governing cytoplasmic viscoelasticity in high-density tissues.

### Intracellular dry mass concentration drives cytoplasmic viscoelasticity during MDCK monolayer maturation

To compute cell dry mass concentration during monolayer maturation, we first renormalized datasets for cell volume *V*_cell_ and dry mass *m*_cell_ with their respective initial subconfluent values (*V*_0_ and *m*_0_) and plotted both quantities against the normalized cell density ρ_cell_/ρ_0_ (Fig. 5A). Relative cell dry mass and volume curves initially decrease in a similar matter at confluence onset (orange data points) by ∼20% (Fig. 5A). However, we noticed a sharper decrease of cell volume (∼41 ± 2%) compared to cell dry mass (∼30 ± 3%) in high–cell density confluent monolayers (red data points) (Fig. 5A). This suggests a rise in intracellular dry mass concentration, which we can estimate to be equal to $\varphi_{\text{intra}}=\frac{m_{\text{cell}}}{V_{\text{cell}}}$ = 0.19 ± 0.02 pg/μm^3^ during the subconfluent regime. Moreover, we quantified intracellular dry mass concentration during cell density increase by first fitting rational functions [$f\left(\rho_{\text{cell}}/\rho_{0}\right)=\frac{1}{\rho_{\text{cell}}/\rho_{0}+A}+B$, with f(1)=1 and *A* and *B* as fitting parameters] to cell dry mass and volume data points (Fig. 5A) and then computing the ratio of both fits ($\varphi_{\text{intra}}/\varphi_{0}=\frac{m\left(\rho_{\text{cell}}/\rho_{0}\right)}{V\left(\rho_{\text{cell}}/\rho_{0}\right)}$) (Fig. 5B). From these results, we conclude that intracellular concentration rises on average by 12 ± 4% as the tissue reaches confluence and further increases by 22 ± 5% in high-density monolayers (Fig. 5B).

**Fig. 5.**
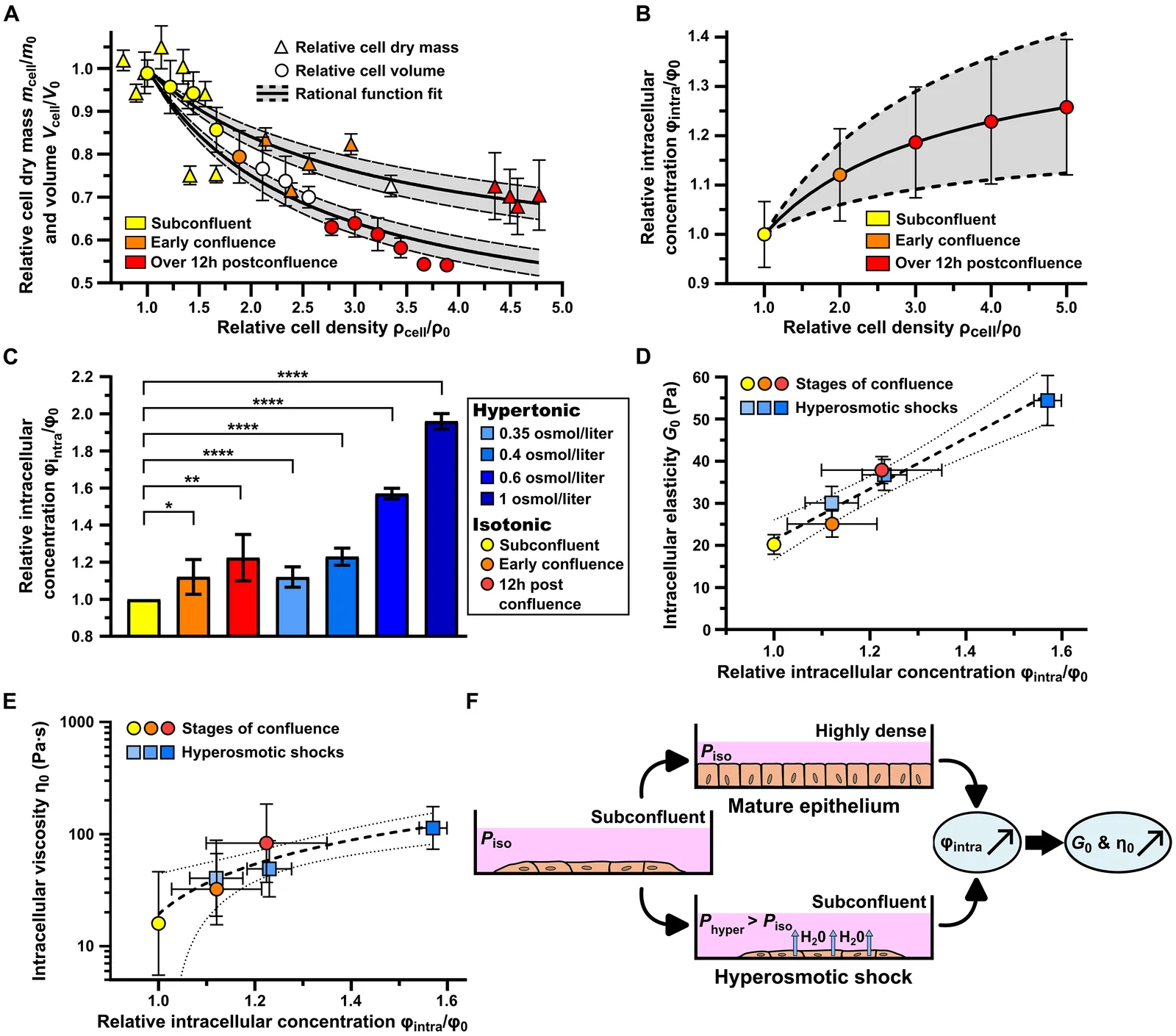
Cytoplasmic viscoelasticity in maturing MDCK monolayers is driven by intracellular dry mass concentration. (**A**) Relative cell dry mass (triangles) and cell volume (circles) as a function of the relative cell density. Normalization for cell dry mass (*m*_cell_), volume (*V*_cell_), and density (ρ_cell_) was obtained by dividing with parameter values in the initial subconfluent regime (*m*_0_, *V*_0_, and ρ_0_). Experimental points are fitted with a linear rational function [$f\left(x\right)=\frac{1}{x+a}+b$, solid lines], forced through the coordinate *x* = 1, *y* = 1. Confidence intervals (95%) for fits are shown in gray. (**B**) Relative intracellular dry mass concentration during cell density increase, obtained from the ratio of rational function fits for cell dry mass over cell volume. Fitting incertitude (95%) and experimental SDs are respectively shown in gray and with error bars. (**C**) Relative intracellular concentration bar plot for MDCK monolayers during different stages of maturation in isotonic medium (yellow to red) and for subconfluent monolayers subjected to various hyperosmotic conditions (blue). The relative intracellular dry mass concentration for hyperosmotic conditions is calculated by considering only the reduction in cell volume. (**D**) Intracellular elasticity and (**E**) viscosity of MDCK cells as a function of relative intracellular dry mass concentration during monolayer maturation (yellow to red) and hypertonic shocks (blue). Experimental data points are fitted with a linear regression (dotted line). (**F**) Schematic representation of the analogy between highly dense monolayers under isotonic conditions and subconfluent monolayers under hypertonic conditions. Both exhibit an elevation in intracellular viscoelasticity caused by an increase of intracellular dry mass concentration. Data points for (A), (B), and (C) correspond to the means ± SD. Data points for (D) and (E) correspond respectively to the means and geometric means with 95% confidence intervals. One-sample *t* test with a null hypothesis value of 1 for (C). ****P < 0.0001; **P < 0.01; *P < 0.05.

Last, to further establish a direct link between cytoplasmic viscoelasticity and intracellular dry mass concentration, we concatenated results obtained in monolayers at different densities with those obtained in subconfluent cells subjected to hypertonic shocks (Fig. 5C). Intracellular concentration inferred upon hypertonic shocks (shades of blue) covers the full range of intracellular concentration obtained during monolayer maturation (yellow to red) (Fig. 5C), with subconfluent islands under 0.4 osmol/liter hypertonic stress (light blue) having an equivalent intracellular dry mass concentration to dense MDCK monolayers in isotonic medium (red) (Fig. 5C). Notably, the cytoplasmic viscoelasticity and intracellular concentration have a remarkably high linear correlation (dotted lines), for tissues at different cell densities or under hypertonic stress (*R*^2^ = 0.98 and *R*^2^ = 0.89, respectively, for elasticity and zero-shear viscosity) (Fig. 5, D and E). While there is a deviation from the linear fit for intracellular viscosity in high-density monolayers (red) (Fig. 5E), these results overall indicate that intracellular dry mass concentration is a key contributor to the increase in cytoplasmic viscoelasticity observed in high-density tissues. To summarize, cell density increase during MDCK monolayer maturation is characterized by a decoupled loss rate of cell volume and dry mass, which leads to an elevated intracellular dry mass concentration driving viscoelastic strengthening of the cell cytoplasm (Fig. 5F).

## DISCUSSION

Our results identify intracellular dry mass concentration as a central physical parameter linking monolayer cell density to cytoplasmic mechanics. By comparing monolayer maturation with acute hypertonic perturbations, we show that similar increases in intracellular concentration, whether achieved through progressive densification at high cell density or rapid osmotic water loss, are associated with comparable rises in cytoplasmic viscoelasticity. This convergence across distinct experimental routes indicates that the cytoplasm’s mechanical state is not directly dependent on cell volume or total dry mass, but on their ratio. As cell density rises in a confluent monolayer, the cell volume decreases faster than its dry mass, thereby increasing intracellular macromolecular concentration, which leads to cytoplasmic stiffening. Such regulation may represent a general mechanism by which epithelial tissues adapt their cytoplasmic material properties during maturation. In particular, the marked increase in cytoplasmic viscosity at high cell density may enhance intracellular force dissipation and reduce the buildup of potentially harmful local stress concentrations. Beyond this adaptive role, the observed decoupling between the evolution of cytoplasmic viscoelasticity and externally measured cell elasticity during monolayer maturation further emphasizes that these measurements reflect distinct mechanical properties of the cell. Different probes interrogate different cellular regions, structures, and timescales and therefore provide complementary information (*69*). The viscosity and elasticity values measured by MRS in this study fall well within the range reported by other active microrheology techniques, such as optical tweezers (table S1). While the measured increase in intracellular viscoelasticity appears to be primarily driven by the rise in cytoplasmic concentration, the decrease in external elasticity likely reflects remodeling of the actin-rich cortex during monolayer maturation. Moreover, the selective reduction in intracellular viscosity following microtubule depolymerization further indicates that cytoplasmic viscoelasticity has a multicomponent origin, arising from the combined contributions of crowding-dependent elastic confinement and cytoskeleton-mediated dissipation and structural relaxation.

These findings also have broader implications for how collective cell states emerge in dense tissues. Increased cytoplasmic viscoelasticity could influence intracellular transport (*18*), mechanotransduction, and the ability of cells to remodel their shape or migrate within crowded environments (*70*, *71*). The strong correlation between concentration and mechanics suggests that physical crowding effects, often described in terms of macromolecular crowding or glass-like cytoplasm behavior (*72*, *73*), may play a major role alongside biochemical regulation. The selective reduction of cytoplasmic elasticity following puromycin treatment suggests that polyribosome complexes contribute notably to intracellular elastic confinement during monolayer maturation, potentially through crowding-induced stiffening of the cytoplasm (*61*). In this view, tissue maturation involves not only changes in gene expression and cell-cell adhesion, but also a gradual shift in the cytoplasm’s physical state. This may help explain why high-density epithelia often display reduced dynamics and enhanced mechanical stability compared with sparse cell populations (*74*). Furthermore, the mechanism by which increasingly dense monolayer tissues adjust their intracellular mechanical properties might also extend to embryogenesis, as previous studies have linked high local cell density within a developing embryo to increased tissue stiffness (*75*, *76*).

While our findings provide a coherent framework linking intracellular concentration to cytoplasmic viscoelasticity, they also open several promising directions for future investigation. The present analysis estimates intracellular concentration from measurements of cell volume and dry mass, an approach that captures global changes but not potential spatial heterogeneities or compartment-specific variations within cells. Future studies combining these measurements with high-resolution mapping techniques could reveal how local cytoplasmic concentration differences contribute to mechanical behavior. Moreover, intracellular concentration variations during hypertonic shocks and long-term monolayer maturation likely involve distinct regulatory responses beyond passive water efflux. While recent data have shown a homeostatic regulation of intracellular dry mass concentration across the cell cycle for proliferating single cells (*77*), the exact mechanism by which cytoplasmic content is adjusted during tissue maturation remains under investigation. Current hypotheses involve the central role of membrane potential in tuning cell volume through the regulation of intracellular osmolyte concentration (*78*), as well as the desynchronization of cell growth and division rates to reach different target intracellular concentrations (*43*).

Future work should therefore aim to directly measure intracellular concentration distributions across tissues, identify molecular components that dominate the viscoelastic response, and test whether manipulating concentration independently of volume can modulate tissue mechanics. Extending these approaches to three-dimensional tissues and living organisms will be essential to establish the physiological relevance of this mechanism. A better understanding of how cells regulate their internal physical state in crowded environments could inform models of tissue development, disease progression, and the design of biomimetic materials that recapitulate mechanical behavior of dense cellular assemblies.

## MATERIALS AND METHODS

### Experimental design

#### Cell culture

MDCK WT type II (ATCC CRL-2936, RRID: CVCL_B034) and MDCK transduced to express free GFP using MISSIONR TRC3 ORF GFP Lentivirus Control (Sigma-Aldrich) were both cultured in incubators at 37°C and 5% CO_2_ using high-glucose Dulbecco’s modified Eagle’s medium (DMEM) with GlutaMAX and pyruvate supplement (Gibco, 31966-021) and 10% fetal bovine serum (FBS; Biowest, S1810-500) + 1% Pen-Strep (Gibco, 15140-122). Cells were regularly washed with 1× phosphate-buffered saline (PBS) and passaged with 0.05% Trypsin-EDTA (Gibco, 25300-054) prior to reaching confluence. Cell cultures were also periodically tested for mycoplasma. For MRS measurements, cells were trypsinized from their T25 flasks after reaching ∼70% confluence and seeded in six bottom-plates (Sigma-Aldrich, TPP 92006) containing sterilized 30-mm glass coverslips (VWR, 631-0174). For each six bottom-plate prepared, cells were seeded to obtain three duplicate samples at different initial seeding densities, to achieve different stages of monolayer maturation (subconfluence, early confluence, and over 12 hours postconfluence). Samples used for MRS were incubated with microwires while subconfluent (1 μl of microwire suspension per culture well), at least 12 hours prior to performing measurements. HeLa GFP cells (RRID: CVCL_0030) used for cell volume method validation (fig. S10) were previously provided by M. Piel’s group and cultured following the same protocol as for MDCK WT cells.

#### Microwire synthesis and characterization

Microwires were synthesized by electrostatic complexation of charged 12-nm maghemite particles as reported by Chevry *et al.* (*52*). Briefly, iron oxide nanocrystals (γ-Fe_2_O_3_) were first coated with poly(sodium acrylate) (*M*_w_ = 2100 g/mol; Sigma-Aldrich, 420344). The negatively charged particles were then redispersed within a prepared 1 M NH_4_Cl solution (Fluka Analytical, 09718). A positively charged polymeric solution of PolyDADMAC (*M*_w_ = 8500 g/mol; Polysciences Inc., 24828) in 1 M NH_4_Cl was separately prepared at the same mass concentration as the nanoparticle dispersion. Coated maghemite particles were then mixed with the polycationic solution to obtain a global charge ratio of −7. The obtained dispersion was dialyzed for 1 hour within a 3-ml 10 K MWCO dialysis cassette (Thermo Fisher Scientific, 66380) under a magnetic field generated by two permanent magnets. Synthesized microwires were washed three times with MilliQ water and sonicated for 45 s to obtain the length and diameter dispersion shown in fig. S3. The sample’s magnetic susceptibility parameter Δχ was obtained by measuring the microwire’s critical frequency ω_C_ in a water-glycerol solution (Sigma-Aldrich, G5516) of known viscosity η_0_ = 237 ± 5 mPa·s (measured with a cone-plate rheometer) (fig. S3).

#### Intracellular viscoelasticity measurements by MRS

MRS experiments were carried out by transferring cells with microwires on glass coverslips, to a PeCon Mini cultivation chamber (POC mini-2, 000000-0441-336). Two ring-shaped silicon spacers (1 and 2 mm width) were added on top of the transferred coverslip. The PeCon chamber was then filled with 1.6 ml of DMEM containing 15 mM of Hepes (Cytiva HyClone 1 M solution, SH30237.01), before sealing it with a sterilized upper coverslip. The closed PeCon chamber was brought under an Olympus IX73 inverted microscope with the MRS device setup (fig. S1). Temperature was maintained at 37° ± 1°C by blowing heated dry air circulated through a thermostatic bath. Temperature control was ensured by sticking a thermocouple probe (Voltcraft, MT-52) to the sample. Both facing pairs of coils were powered by two electrical signals with a 90° phase shift generated with a multifunction generator (RIGOL DG952), to give rise to a 12-mT rotating magnetic field. The field was measured with a gaussmeter (Hirst Magnetic Instrument, GM08) to verify its homogeneity and magnitude in a 1.5-by-1.5 mm square region at the center of the MRS device. Electrical inputs were visualized with an oscilloscope (RIGOL, MSO5104) at all times. Cell monolayers were observed using a 60× Olympus phase objective (LUCPFLN60XPH/0.7), and time lapses were acquired with a CCD camera (QImaging, EXi Blue) piloted by the Metamorph software. For each field of view, time lapses were acquired at nine distinct rotation frequencies (150, 84, 47, 27, 15, 8.4, 4.7, 2.7, and 1.5 mHz) to determine the wire’s critical frequency ω_C_, used to compute intracellular zero-shear viscosity. The duration of these time lapses was adjusted to ensure at least one to two full wire rotation in the synchronous regime. Noninternalized microwires often slightly out of focus and showing no asynchronous to synchronous transition in the frequency range probed by MRS were excluded from the analysis (movie S5). Intracellular elastic modulus at high frequency was determined by performing two bright-field stream acquisitions of microwires under a 10-Hz rotating magnetic field. In the context of MRS measurements during monolayer maturation, the increase in cytoplasmic viscoelasticity at high cell density was not attributed to an apparent confinement effect of microwires (fig. S8). MRS quantifications were restricted to microwires with lengths, which did not show a significant correlation (Spearman) with intracellular viscoelasticity in low– and high–cell density monolayers (2 to 5 μm for viscosity measurements and 2 to 7 μm for elasticity measurements) (fig. S8). Moreover, we observed no significant correlation of intracellular viscoelasticity measurements in subconfluent monolayers with the microwire’s localization relative to the cell membrane (fig. S9, A to C). While a slight increase of intracellular viscosity was observed for microwires close to the cell nucleus (fig. S9E), this modest rise is not sufficient to explain the substantial increase measured in dense confluent monolayers, thus excluding nuclear confinement artifacts (fig. S9F). Density categories (subconfluent, onset of confluence, over 12 hours postconfluence) for MRS measurements have an incertitude on time as the protocol takes 2 to 3 hours to implement, given the large number of low frequencies probed.

#### MRS data analysis

The zero-shear viscosity η_0_ at low frequency (150 to 1.5 mHz) can be computed from the previously derived equation assuming a Maxwellian fluid model (*47*, *52*): $\eta_{0}=\frac{3}{8}\frac{\mu_{0}∆\chi }{\omega_{c}}g\left(\frac{L}{D}\right)\frac{D^{2}}{L^{2}}H^{2}$, where *L* and *D* are respectively the microwire’s length and diameter, Δχ its magnetic susceptibility parameter, ω_C_ is the microwire’s critical frequency separating the synchronous and asynchronous regime, μ_0_ is the vacuum permeability, *H* is the magnetic field amplitude (12 mT), and $g\left(\frac{L}{D}\right)=\text{ln}\left(\frac{L}{D}\right)-0.662+0.917\frac{D}{L}-0.05{\left(\frac{D}{L}\right)}^{2}$ is the dimensionless function for a rigid rod valid for an anisotropy ratio $\frac{L}{D}$ comprising between 2 and 30 (*79*). The elastic modulus *G*_0_ at high frequency is obtained from an analogous equation: $G_{0}=\frac{3}{4}\frac{\mu_{0}∆\chi }{\theta_{0}}g\left(\frac{L}{D}\right)\frac{D^{2}}{L^{2}}H^{2}$ where θ_0_ is the wire’s amplitude of oscillation in the asynchronous regime at 10 Hz. Lengths and diameters were measured manually on ImageJ for each microwire. Critical frequencies ω_C_ were determined by visualizing time lapses acquired at various low frequencies for different microwires (between 150 and 1.5 mHz). The angle of oscillation θ_0_ at 10 Hz was measured manually on ImageJ and averaged over at least four data points on two separate acquisitions, to ensure a stable representative value. In practice, only microwires with a critical frequency ω_C_ at least 500 times below 10 Hz were considered for elastic modulus measurements.

#### External cell elasticity measurements by nanoindentation

Cell monolayers cultured on Fluorodish (WPI, FD35-100) were brought under an inverted microscope set with the Chiaro nanoindenter system (Optics11 Life). Prior to measurements, the nanoindentation probe (27.5 μm radius and 0.02 N/m stiffness, Optics11 Life P230647M) was calibrated with an integrated standard protocol on the commercial Piuma software. Measurements were carried out on cell monolayers by imposing cantilever displacement (5-μm indentations at 10 μm/s with 1-s holding time). Load-displacement graphs were fitted with the Piuma software to recover the sample’s effective Young’s modulus. Measurements were performed on cells with different spreading areas located throughout the monolayer, to eliminate any potential biases linked to cell morphologies.

#### Cell dry mass measurements by QPI

Due to low pattern stability on glass, samples were prepared by seeding MDCK cells at different initial densities on 1-mm-diameter disk-shaped fibronectin patterns in glass bottom Fluorodishes, so as to match cell spreading areas obtained for MRS experiments (Figs. 3B and 2B). Briefly, a polydimethylsiloxane (PDMS) mold containing several 1-mm-diameter cylinders was incubated with 200 μl of fibronectin (50 μg/ml, Sigma-Aldrich, F1141) for 45 min. Fluorodishes were plasma cleaned for 10 min before being stamped with fibronectin-coated PDMS patterns. The glass surface surrounding fibronectin patterns was passivated with PLL-g-PEG solution (0.1 mg/ml; SuSoS, SZ34-67) for 2 hours before cell seeding. MDCK cells were then grown for 48 hours prior to QPI measurements, which were obtained using a custom-built thin diffuser-based wavefront sensor (*80*, *81*). Cells were illuminated in transmission with an LED (λ = 635 nm, Δλ = 10 nm) placed in a Köhler illuminator, ensuring high spatial coherence (*NA*_ill_ 0.05). Images were acquired with a ×20 microscope objective (*NA* = 0.4) and recorded on a camera (PCO Panda), in front of which a thin holographic diffuser (Edmund Optics, no. 47-991) was placed in close proximity (*d* = 2.6 mm) to the sensor. Quantitative phase maps were obtained by numerical integration of the local speckle displacements. These maps were then converted into dry mass density maps assuming a specific refractive index increment of 0.19 μm^3^/pg. The total dry mass was then obtained by simply integrating the images over the area of the imprinted disk. Cell density within each pattern was determined by manual counting on ImageJ, and the mean dry mass per cell was estimated by dividing the total pattern dry mass by the number of cells. Cells in areas of high extrusion found in dense monolayer samples were taken into account by first counting cells in surrounding monolayer regions (with no extrusions), computing cell density, and then multiplying extrusion areas by twice the cell density (as high signals for dry mass surface density in dense monolayers corresponded to twice the optical path difference found in surrounding monolayer regions).

#### Cell volume measurements (monolayer maturation and osmotic shocks)

For volume measurements on maturing monolayers, MDCK free cytosolic GFP cells were seeded and incubated on glass-bottom Fluorodish with Red Phenol–free culture medium, until obtaining a monolayer at 70 to 80% confluence. Samples were then brought under a Zeiss LSM980–Airyscan2 with an incubator chamber set at 37°C and 5% CO_2_. For each replicate experiment, four to six subconfluent fields of view were selected. *Z*-stacks with 0.32-μm steps were acquired every hour with a Zeiss 40 × 1.2 ImmKorrDIC(UV)VIR-IR Multiimmersion objective (configured for water), until at least 15 hours postconfluence. The data were preprocessed using ZenBlue software. Acquired *z*-stack time lapses were then analyzed using a custom written thresholding algorithm (*18*). Average cell volume was estimated by dividing the evolution of total cell volume within each field of view with the number of cells, determined by manual counting on ImageJ. Volume measurements for hypertonic shocks were conducted on subconfluent MDCK cell islands (20 to 30% confluence). Relative cell volume change during hyperosmotic stress was obtained by acquiring *z*-stacks before and after the shock and by using the previously described workflow. All data points for cell volume determination during osmotic stress were renormalized by the control condition (replacing with fresh isotonic medium). This experimental method was validated by performing additional measurements on HeLa GFP cells subjected to two distinct hyperosmotic conditions (0.45 and 0.6 osmol/liter) and comparing these results to data points obtained by Venkova *et al.* (*82*) who used the fluorescence exclusion microscopy protocol (fig. S10)

#### Cell velocity, cell area, and MSD quantification

After carrying out MRS measurements on monolayers at different cell densities, 1-hour time lapses (12 frames) were acquired with a 20× phase objective (Olympus, LCAchN20XPhC/0.4) to determine the mean cell velocity with PIVlab. A standard two-pass (64 and 32 pixels, i.e., 20.4 and 10.2 μm) FFT window deformation method was implemented, and the data were processed by filtering out outlier velocity vectors. Mean cell velocity was computed by taking the spatiotemporal average of velocity magnitude |*v*|. Mean cell velocity decrease in maturing monolayers (Fig. 2B) did not jam to 0 μm/hour, as the window size chosen for PIV measurements was likely small enough to also detect intracellular motion. Cellpose4 CPSAM model was used for segmentation to determine mean cell area in different experimental conditions. The MSD of cells during monolayer maturation was calculated by averaging hundreds of individual tracks obtained on TrackMate.

#### Wound healing assay

Wound healing assays for MRS measurements were prepared by seeding MDCK cells in two-well culture inserts (Clinisciences, 80209-150) and incubating monolayers until maturation onset, over 12 hours postconfluence. The culture medium was renewed daily to ensure proper cell growth. When dense monolayers were obtained in both confinement wells, the insert was removed, and the glass coverslip was transferred to an enclosed PeCon cultivation chamber. A first round of MRS measurements was carried out at the monolayer’s edge and bulk for a maximum of 3 hours. The sample was then left to migrate overnight (15 hours) and a 12-frame per hour time lapse was acquired with a 10× phase objective (CPlanFLN10XPhC/0.3) for cell velocity measurements by PIV. The observed rise in mean cell velocity at the edge (fig. S5B) corresponds to the progressive increased proportion of motile cells in the region of interest, as the monolayer spreads. Eighteen hours post–insert removal, additional MRS measurements of intracellular viscoelasticity were acquired at the monolayer’s bulk and migrating edge for a maximum of 3 hours. Following MRS measurements, the PIV workflow described in the previous section was applied to extract mean cell velocity at the monolayer’s edge (below 150 μm from edge) and bulk (above 150 μm from edge).

#### Cell cycle determination by fluorescence-activated cell sorting

Monolayers were grown to different cell densities in T25 flasks, and the time relative to confluence was determined (for subconfluent samples, this time was estimated by tracking the evolution of replicate flasks). Cells were then trypsinized and resuspended in 600 μl of medium, before being fixed with a 70% ethanol solution at −20°C. Samples were washed with fluorescence-activated cell sorting (FACS) buffer (2% FBS and 1 mM EDTA) and stained with propidium iodide (50 μg/ml; Sigma-Aldrich, 81845), RNase (100 μg/ml; Roche, 10109169001), and 2 mM MgCl_2_ in PBS for 20 min at room temperature. After filtration, the cell cycle was analyzed by flow cytometry using an Attune CytPix (Thermo Fisher Scientific). Propidium iodide fluorescence was detected following laser excitation at 561 nm, and emission was collected using a 620/15 band-pass filter. Flow cytometry data were processed using FlowJo software (version 10). Cell cycle phase distribution of G1/G0, S, and G2 was determined using the Watson pragmatic model.

#### Osmotic perturbations

Hypertonic shock solutions at 0.35, 0.4, 0.6, and 1 osmol/liter were prepared by respectively adding 50, 100, 200, or 300 mg of sucrose (Sigma-Aldrich, S0389-500G) to 3 ml of DMEM (0.3 osmol/liter). Hypotonic shock solution at 0.1 osmol/liter was simply obtained by diluting cell culture medium to 1/3 with MilliQ water. MRS experiments were carried out for a maximum of 2 hours after shock induction.

#### Drug perturbations

Prior to MRS or nanoindentation experiments, subconfluent MDCK samples were incubated for 30 min in all drug perturbation experiments (cytochalasin D, Sigma-Aldrich C8273; latrunculin A, Sigma-Aldrich L5163; puromycin, Gibco A1113803), with the exception of microtubule disruption (nocodazole, Thermo Fisher Scientific 358240100) where the incubation time was 1 hour 30 min. The following concentrations for different drugs were chosen in accordance to previous studies (*61*, *83*, *84*), to ensure a substantial disruption of the cell cytoskeleton and polyribosome complex: 4 μM for actin depolymerization drugs (cytochalasin D and latrunculin A), 33 μM for microtubule disruption with nocodazole, and 100 μg/ml for polysome collapse by puromycin treatment. The effect of polysome collapse on intracellular viscoelasticity was also tested in highly dense monolayers, by performing MRS experiments before and 30 min after puromycin addition.

#### Cell staining

Live stainings of cell membranes in subconfluent MDCK monolayers with internalized microwires were carried out by incubating samples with 200 nM Lipilight MemBright Cy5 (640 nm, MC0MEM6401902) in culture medium without FBS for 10 min. Stainings of the actin cytoskeleton, microtubules, and nuclei were performed on samples fixed with 4% PFA (Thermo Fisher Scientific, 28908) at 37°C for 15 min. Fixed samples were washed six times with 1× PBS, and membranes were permeabilized using 0.5% Triton (Sigma-Aldrich, T8787) for 15 min. Following permeabilization, samples were blocked with a solution of 3% bovine serum albumin (Roche, 10735094001) and 10% FBS in PBS for 2 hours. Microtubules were stained by immunofluorescence through overnight incubation with α-tubulin primary antibody (Sigma-Aldrich, catalog no. T9026, RRID: AB_477593), followed by 2-hour incubation with a secondary antibody solution (Thermo Fisher Scientific, catalog no. A-11001, RRID: AB_2534069) with 10% FBS. The actin cytoskeleton was stained with Phalloidin-Alexa568 for 2 hours (Life Technologies, A12380) and nuclei with DAPI Hoechst for 30 min (Invitrogen, H3570). Stained samples were mounted with Mowiol 4-88 for imaging (Sigma-Aldrich, 81381-250G).

### Statistical analysis

Statistical tests and fits were computed on GraphPad Prism version 8.02. “*N*” describes biological replicates while, “*n*” represents the total number of independent measurements. All data points are represented with their mean values and standard deviations, at the exception of intracellular viscosity measurements, which are plotted with their geometric means and geometric standard deviations (log normal distribution). Standard error of the mean and geometric standard error of the geometric mean are used to report incertitude on data in the main text. Mann-Whitney tests were performed to compare non-Gaussian datasets, whereas Student’s *t* test and Welch’s *t* test were applied to Gaussian datasets with respectively equivalent and significantly different standard deviations. Fits (linear and rational functions) are shown with their 95% confidence interval.

## Supplementary Materials

### The PDF file includes:

Figs. S1 to S10 Table S1 Legends for movies S1 to S5 References
