# Supplementary material for: Cytoplasmic crowding drives intracellular viscoelastic increase during epithelial maturation
Source: Sci Adv. 2026 Nov 4;12(45):eaeh7786. doi: 10.1126/sciadv.aeh7786 (PMC13733821; doi:10.1126/sciadv.aeh7786)
Supplement: Supplementary file 1 [file sciadv.aeh7786_sm.pdf]

Science Advances
AAAS

Supplementary Materials for

Cytoplasmic crowding drives intracellular viscoelastic increase during epithelial maturation

Gregory Arkowitz *et al.*

Corresponding author: René-Marc Mège, rene-marc.mege@ijm.fr; Jean-François Berret, jean-francois.berret@u-paris.fr; Benoit Ladoux, benoit.ladoux@fau.de

Sci. Adv. **12**, eaeh7786 (2026)
DOI: 10.1126/sciadv.aeh7786

The PDF file includes:

Figs. S1 to S10
Table S1
Legends for movies S1 to S5
References

Other Supplementary Material for this manuscript includes the following:

Movies S1 to S5

Figure S1:

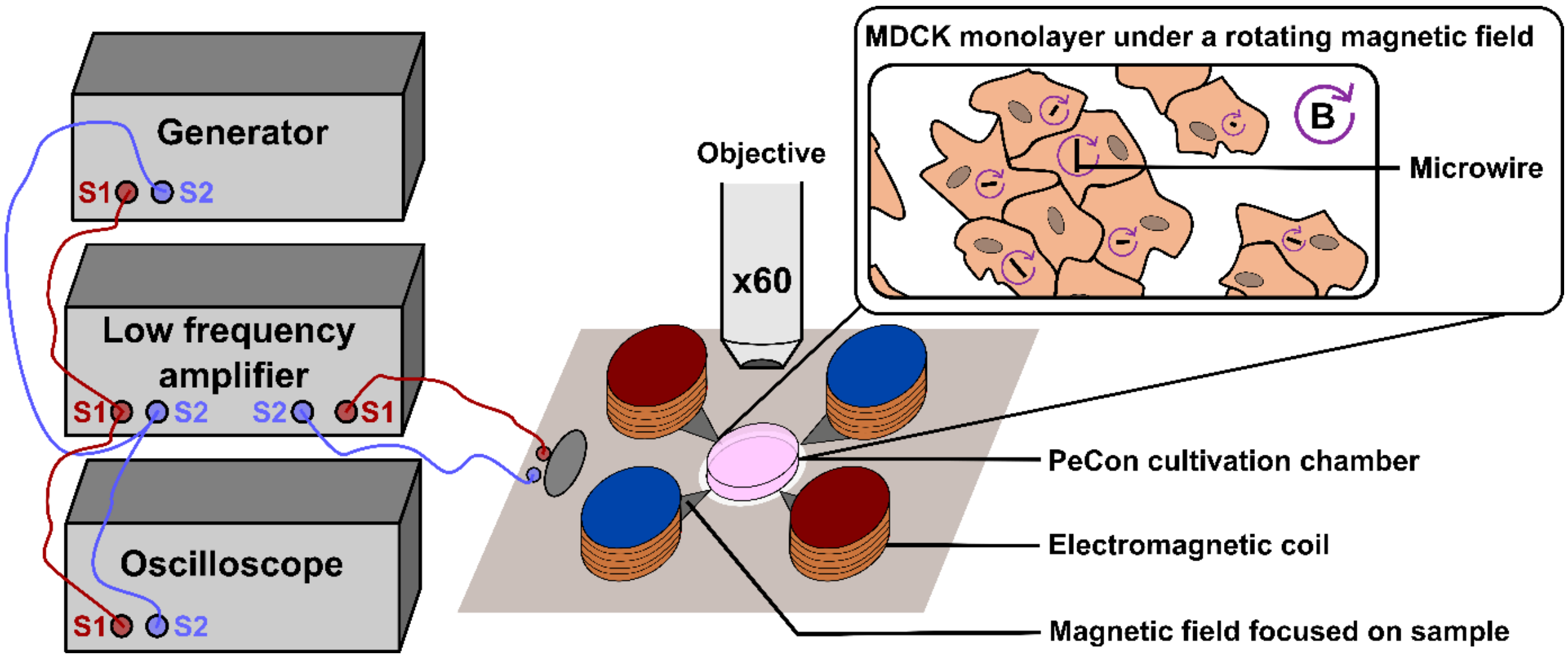

Fig. S1. Magnetic rotational spectroscopy (MRS) experimental setup

Experimental setup schematic for magnetic rotational spectroscopy (MRS). A PeCon cultivation chamber containing MDCK cells with internalized microwires is placed at the center of two pairs of electromagnetic coils (blue and red). These pairs of coils are powered with two electrical signals (S1 and S2) with a 90° phase shift, allowing for the establishment of a rotating homogeneous magnetic field $\vec{B}$ focused radially towards the center. Input signals are produced by a generator in series with a custom-built low frequency amplifier and are monitored at all times with an oscilloscope. Cells and microwire dynamics are visualized by phase contrast and brightfield microscopy.

Figure S2:

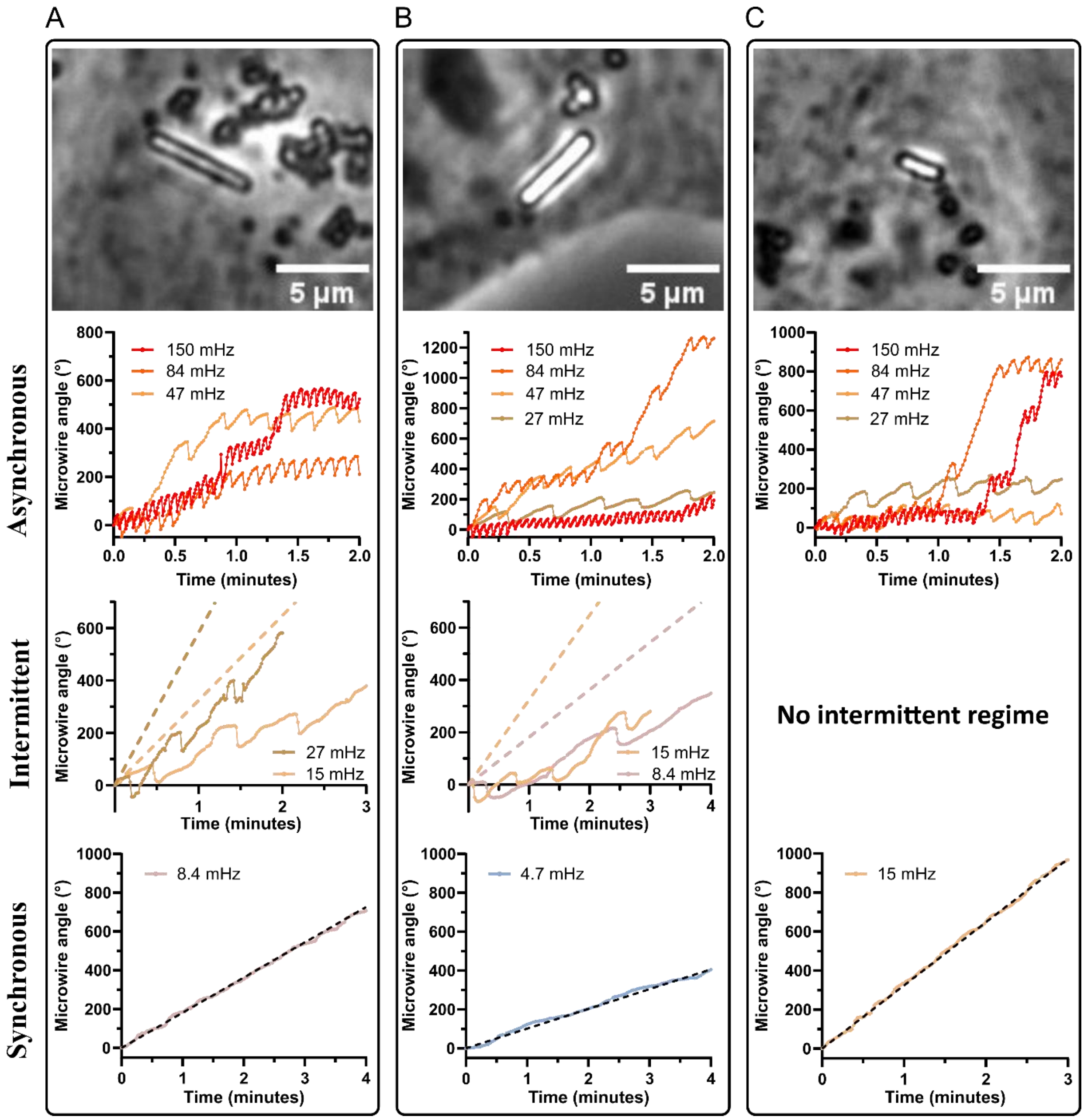

Fig. S2 Microwire asynchronous to synchronous dynamic transition during magnetic field frequency sweeping

Tracking profiles of three microwires (**A**), (**B**) and (**C**) subjected to a magnetic field rotating at various frequencies (from 150 mHz to 4.7 mHz). These profiles were obtained with the ImageJ “Directionality” plugin applied to cropped timelapses of highly contrasted microwires shown in phase contrast at the top of the figure. As the frequency decreases microwires go from an asynchronous motion (top row), to an intermittent regime with regular oscillations (middle row), to a final synchronous regime with no oscillations (bottom row). Dotted lines for intermittent and synchronous regime show the magnetic field angle speed at the frequencies indicated.

Figure S3:

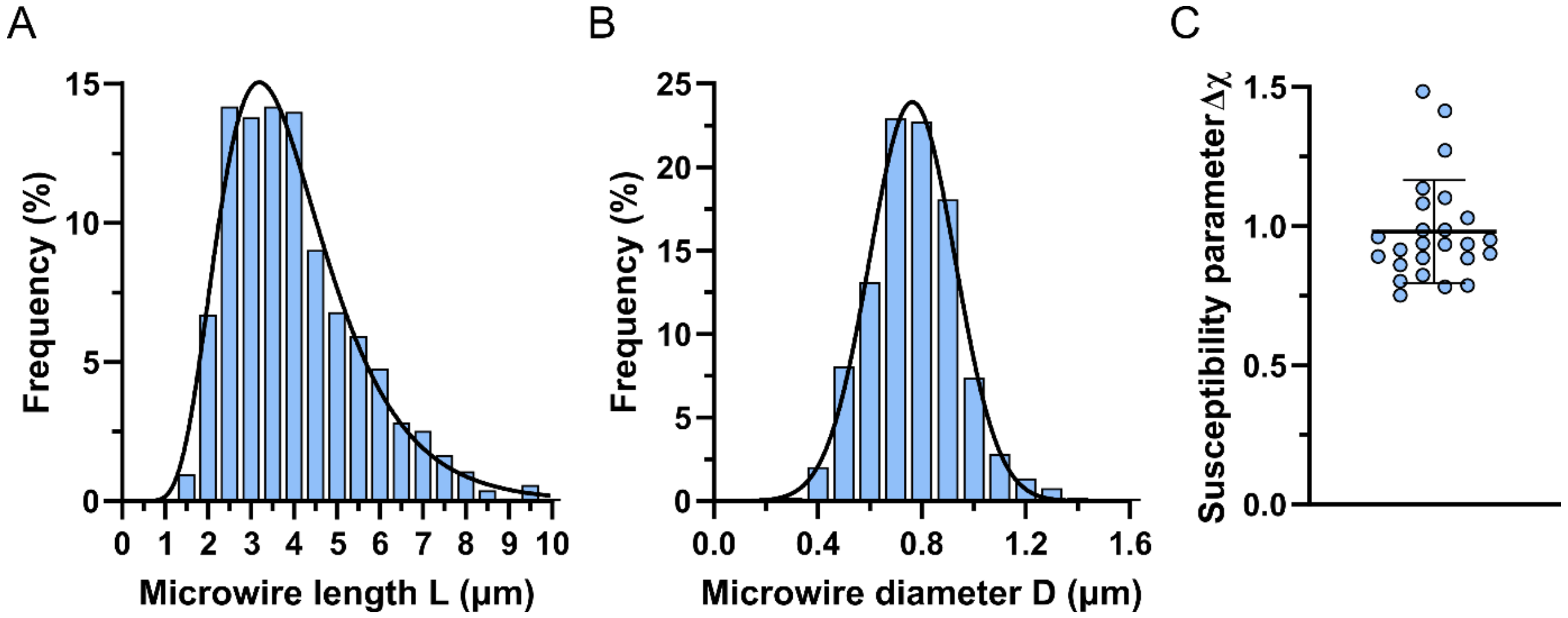

Fig. S3. Microwire geometric and magnetic properties

Microwires' length (**A**) and diameter (**B**) histograms for all MRS experiments on MDCK monolayers (n=1030, N=53), binned respectively with 0.5 µm and 0.1 µm bins. Length distribution was fitted with lognormal function, gaussian function for diameter distribution (black line). (**C**) Microwires' susceptibility parameter $\Delta\chi$ (n=25, N=1), measured in a purely viscous water-glycerol solution of known viscosity η_0 = 237 mPa.s ± 5 mPa.s. The obtained mean susceptibility (represented with its standard deviation) was used to compute intracellular viscoelasticity for all experimental conditions.

Figure S4:

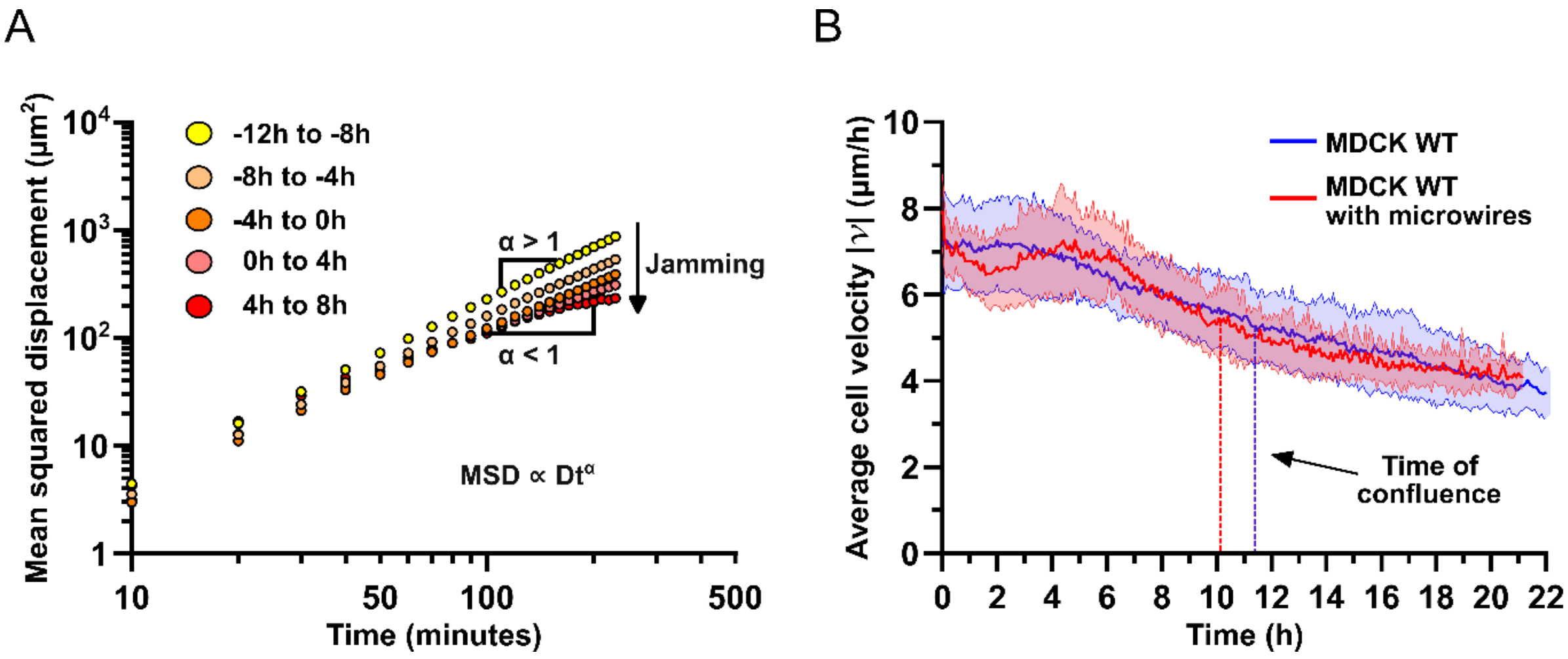

Fig. S4. Monolayer jamming at early confluence

(**A**) Mean squared displacement (MSD) of MDCK cells through time, as the monolayer becomes confluent. MSD is averaged on over 80 cell tracks for 21 different fields of view and is divided into 4h bins (from yellow to red). The powerlaw exponent α is computed for each 4h timeframe by fitting a nonlinear function with two adjustable parameters to MSD curves ($MSD = 10^{\alpha*\log(t)+\beta}$) (**B**) Average cell velocity through time for an MDCK monolayer transitioning from a subconfluent to a fully confluent state, with microwires (red, n=9, N=1) and with no microwires (blue, n=22, N=1). The average cell velocity corresponds to mean velocity magnitude |v| computed by PIV for different fields of view. Standard deviations are shown in lighter colors. Vertical dotted lines indicate the average time of confluence for both experimental conditions.

Figure S5:

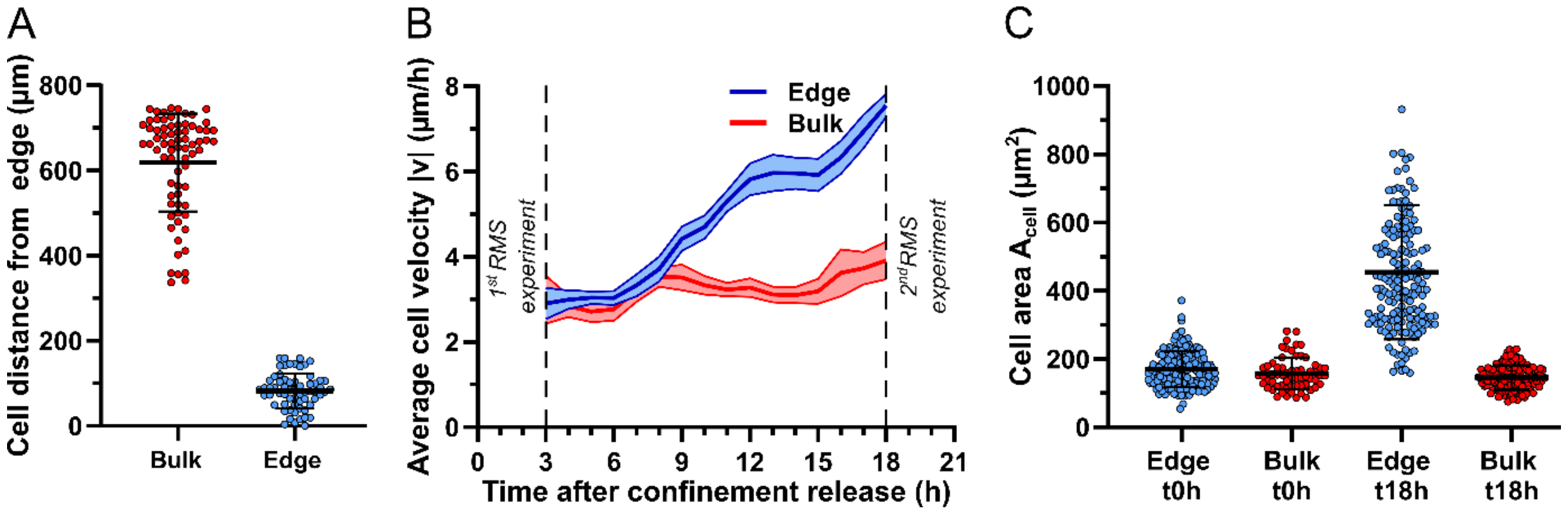

Fig. S5. Monolayer unjamming post-confinement removal

(**A**) Distance from the migrating edge for cells characterized by MRS, at the monolayer edge (n=72, N=4) and bulk (n=63, N=4). (**B**) Average cell velocity up to 18h post-confinement release, at the monolayer edge (blue, n=2, N=2) and bulk (red, n=2, N=2). The average cell velocity corresponds to mean velocity magnitude computed by PIV within each region of interest (above and below 150 µm from the monolayer's edge). Standard deviations are shown in lighter colors. (**C**) Cell area quantifications (CellPose) at the monolayer's edge and bulk, upon confinement removal and 18h post-release (n≥63, N=4). Box plots for all panels show averages and standard deviations.

Figure S6:

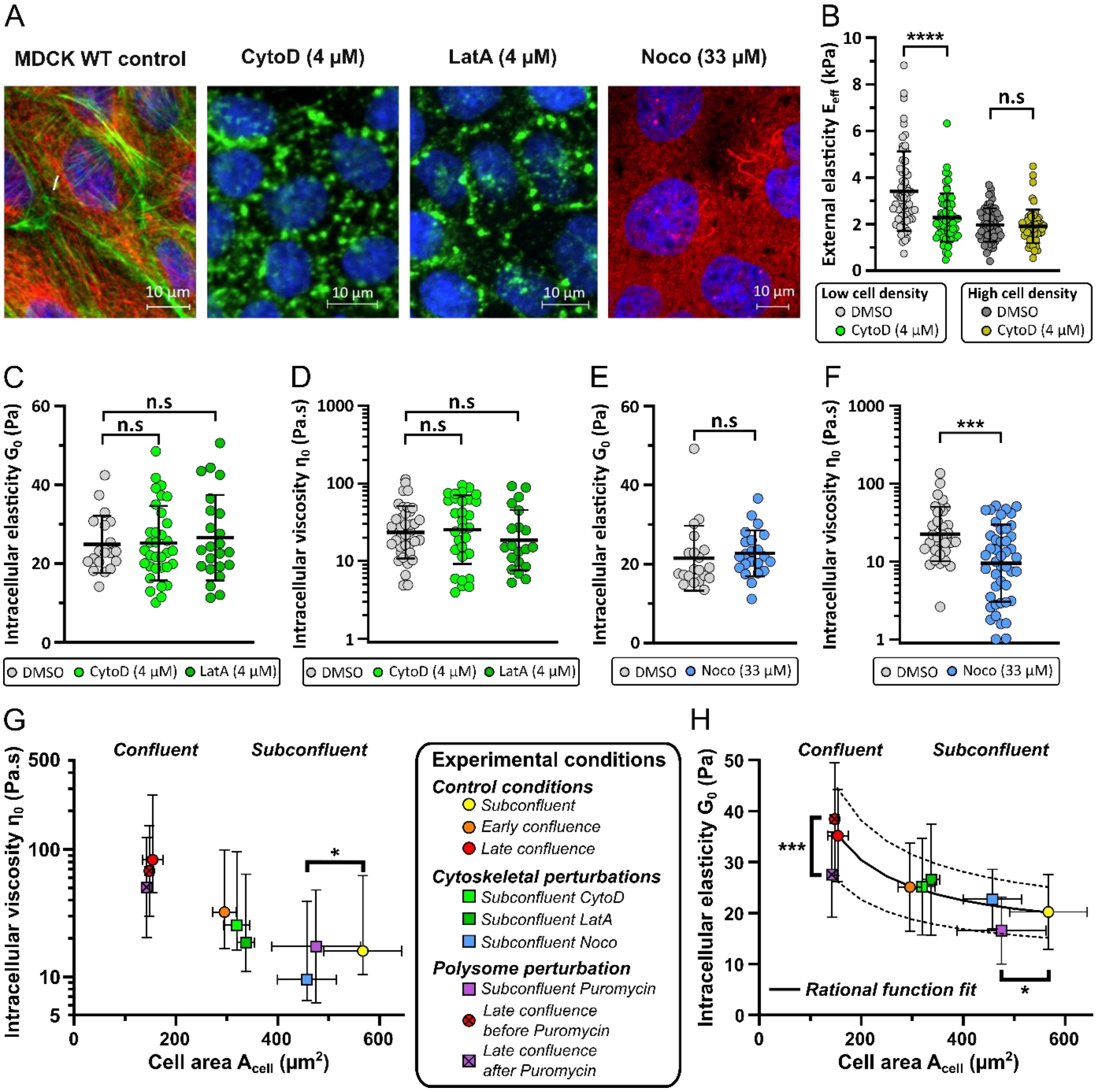

Fig. S6. Impact of drug perturbations on external elasticity measured by nanoindentation and intracellular viscoelasticity measured by MRS

(**A**) Fixed subconfluent MDCK samples stained for actin (green), microtubules (red) and nuclei (blue). Staining images show a mesh-like structure for actin and microtubules in the control condition (MDCK WT control). Cells treated for 30 minutes with Cytochalasin D or Latrunculin A (CytoD 4 µM, LatA 4 µM) exhibit strongly disrupted actin networks. 90 minutes Nocodazole incubation (Noco 33 µM) leads to near-complete depolymerization of microtubules. Scale bar 10 µm. (**B**) External cell elasticity in subconfluent (light green, n=60, N=2) and highly dense confluent monolayers (olive green, n=60, N=2) under 4 µM Cyto D, along with their associated DMSO controls (light grey and dark grey, n=60, N=2). (**C**) Intracellular elasticity (n≥19, N=2) and (**D**) viscosity (n≥20, N=2), for subconfluent monolayers subjected to 4 µM Cyto D (light green) or 4 µM Lat A (dark green), compared to DMSO control (light grey). (**E**) Intracellular elasticity (n≥20, N=2) and (**F**) viscosity (n≥33, N=2), for subconfluent monolayers incubated with 33 µM Noco (blue) or in DMSO control (light grey). (**G**) Intracellular viscosity and (**H**)

elasticity plotted as a function of cell area for different drug perturbation experiments, compared to MDCK monolayers at subconfluence (yellow, n≥39, N=7) early confluence (orange, n≥30, N=3) and late confluence (red, n≥23, N=3). Cytoskeletal perturbations on subconfluent monolayers involve actin disruptions with Cyto D (light green, n≥30, N=2) or Lat A (dark green, n≥20, N=2), as well as microtubule depolymerization with Noco (blue, n≥22, N=3). Polysome perturbations with 100 µg/mL puromycin on subconfluent monolayers (purple, n≥24, N=2). MRS measurements were also acquired before (red with a cross, n≥30, N=2) and after (purple with a cross, n≥28, N=2) addition of 100 µg/mL puromycin in dense confluent monolayers. The dataset with no drug perturbation for intracellular elasticity (yellow, orange and red) was fitted with a rational function (black solid line, $G_0 = \frac{F}{Cell\ area} + G^*$ with F = 3.2 ± 0.9 nN and G* = 14.5 ± 3.5 Pa, corresponding to fitting parameters associated respectively with typical cell force and intracellular elasticity magnitudes), represented with 95% CI (dotted lines). Mean values with standard deviation for panels (**B**), (**C**), (**E**) and (**H**). Geometric mean with geometric standard deviation for panels (**D**), (**F**) and (**G**). Mann-Whitney tests for panels (**B**) and (**E**). Unpaired t-tests for panels (**C**) and (**H**). Unpaired t-tests on log values for panels (**D**), (**F**) and (**G**). **** P<0.0001, *** P<0.001, * P<0.05, n.s P≥0.05.

Figure S7:

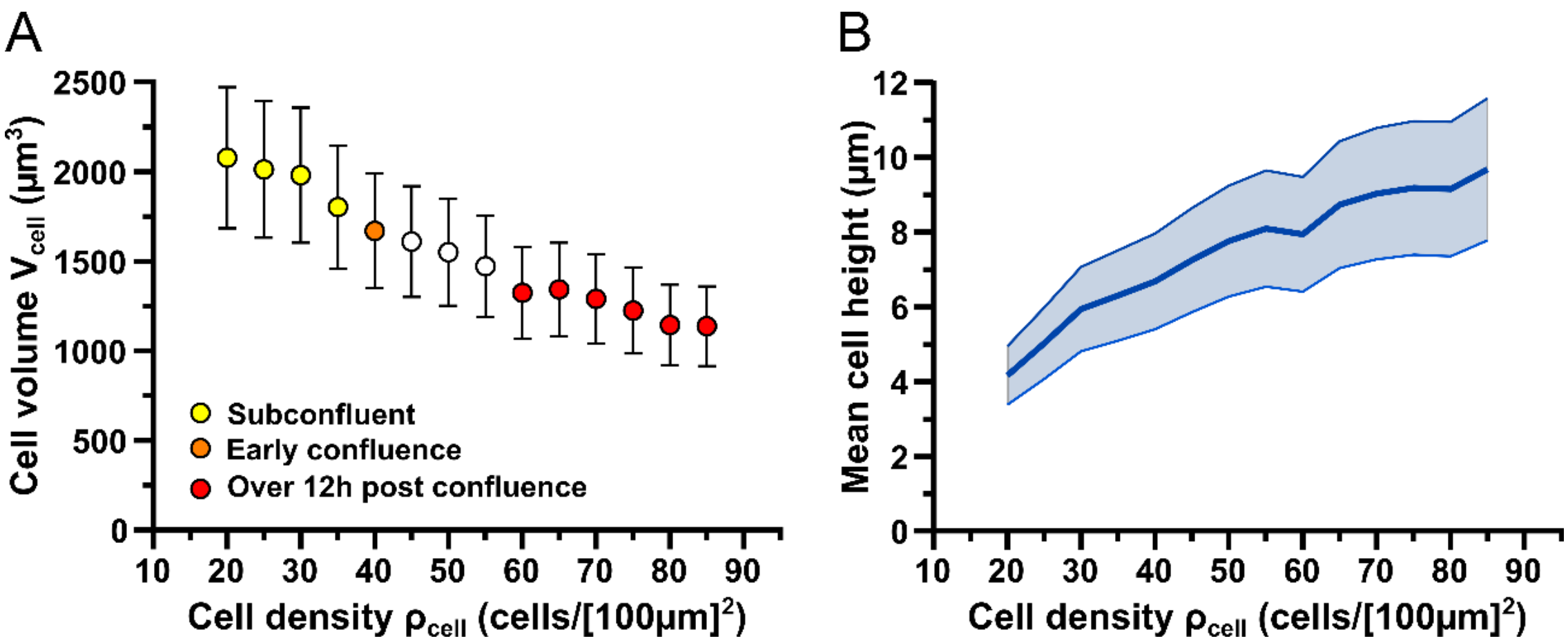

Fig. S7. Cell volume and height evolution during monolayer maturation

(**A**) Cell volume V_{cell} as a function of cell density ρ_{cell} within a progressively maturing monolayer, from subconfluence (yellow) to dense confluent monolayer (red). This curve is the result of multiplying the evolution of relative cell volume during monolayer maturation (Fig. 3H) with the mean cell volume measured in subconfluent layers (Fig. 3G). The standard deviation was calculated by propagation of uncertainty. (**B**) Average cell height increase as the monolayer becomes denser from a subconfluent state (~15h pre-confluence) to a fully confluent monolayer (~24h post-confluence). The height is estimated by dividing mean cell volume V_{cell} with average cell area A_{cell} (standard deviation, light blue).

Figure S8:

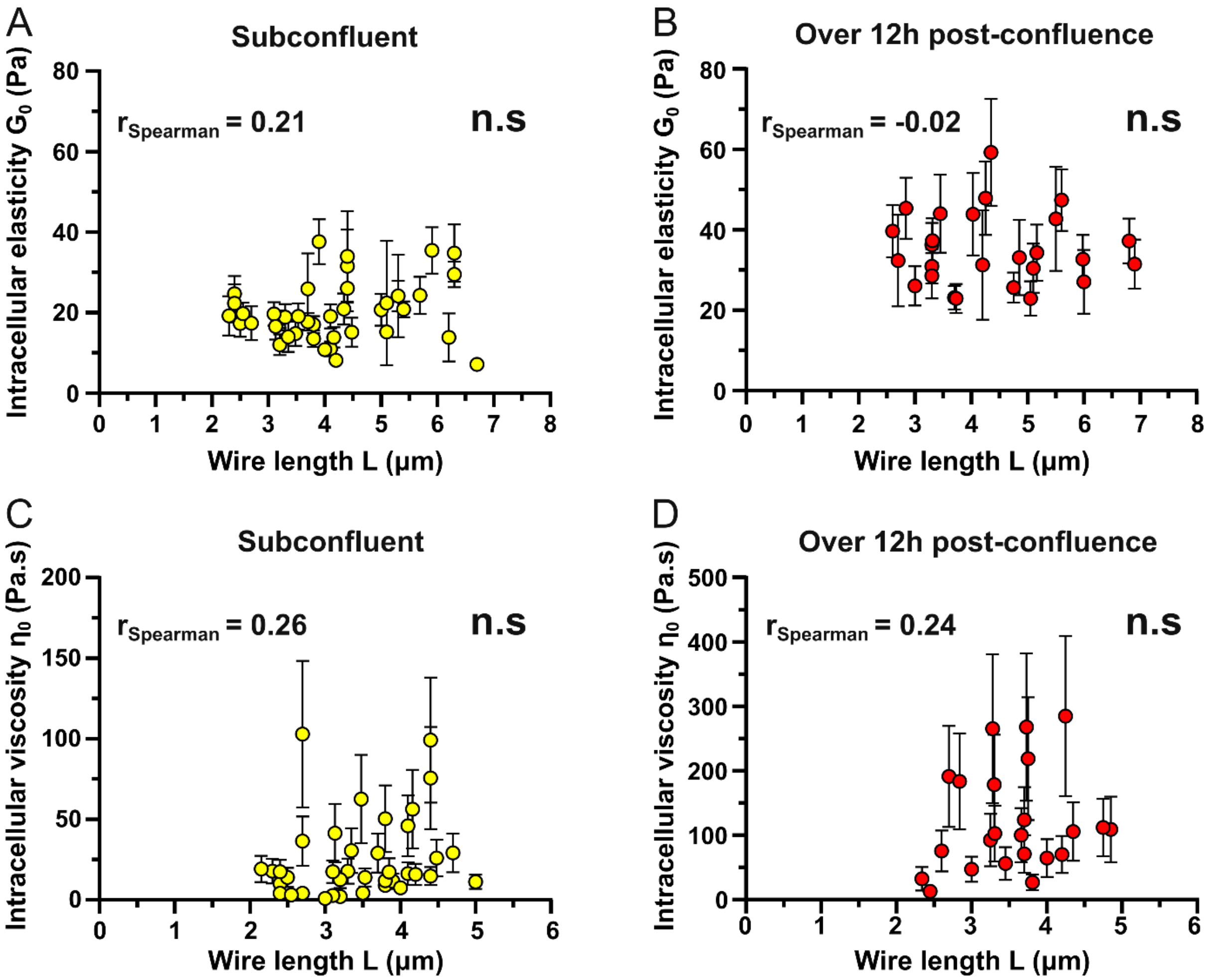

Fig. S8. Intracellular viscoelasticity dependence on microwire length

Intracellular elasticity in (**A**) subconfluent (n=41, N=7) and (**B**) highly dense monolayers (n=26, N=3), plotted as a function of microwire length. Spearman's correlation coefficient is non-significant (P≥0.05) for both density conditions, within the selected microwire length range (2-7 µm). Intracellular viscosity in (**C**) subconfluent (n=39, N=7) and (**D**) highly dense monolayers (n=23, N=3), plotted as a function of microwire length. Spearman's correlation coefficient is non-significant (P≥0.05) for both density conditions, within the selected microwire length range (2-5 µm). Error bars correspond to the computed propagated measurement uncertainty for each microwire.

Figure S9:

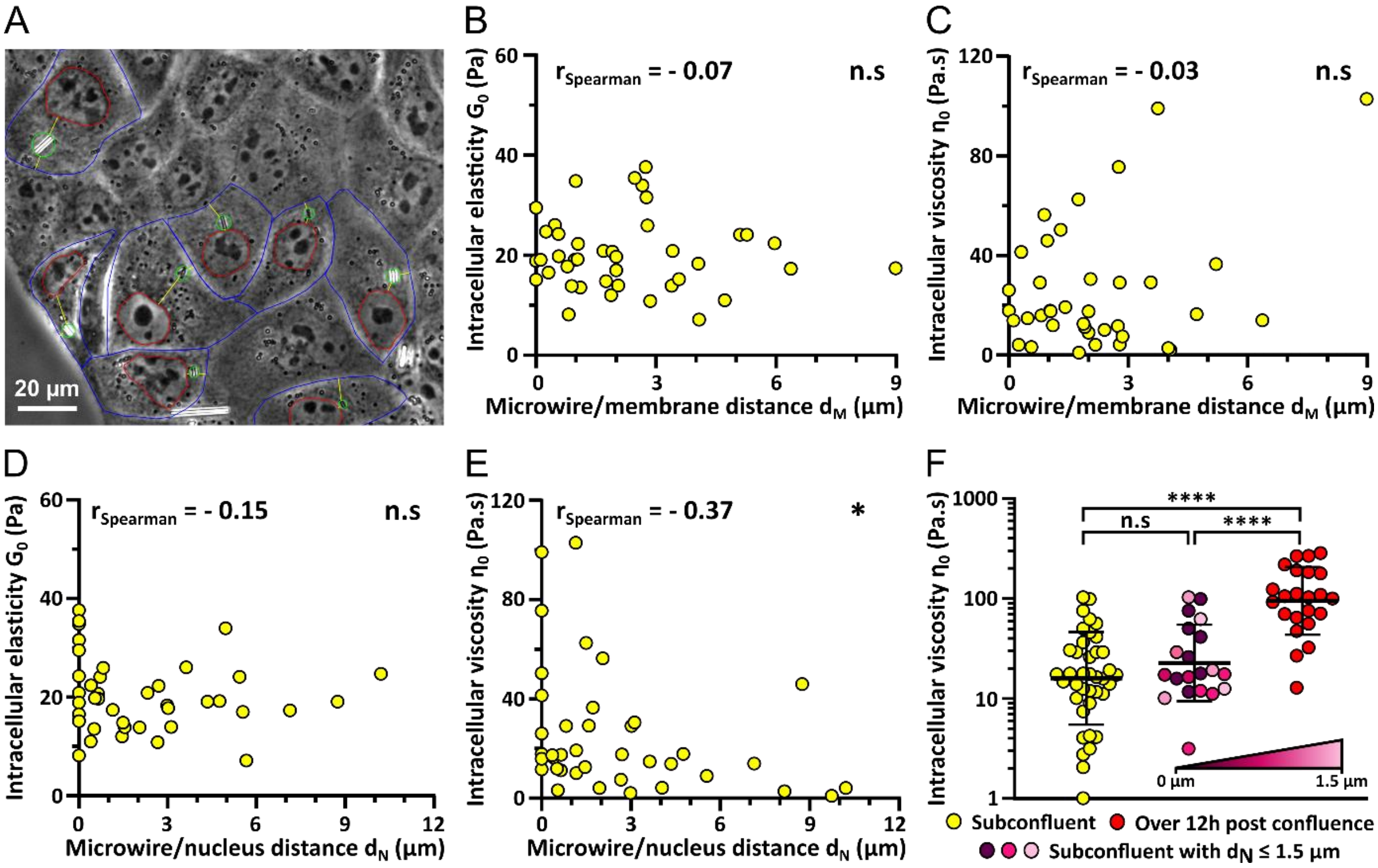

Fig. S9. Intracellular viscoelasticity dependence on microwire localization relative to the cell membrane/nucleus

(**A**) Subconfluent MDCK cells with internalized microwires, from (**Fig. 1C**). Microwires used for MRS quantification are encircled (green). Cell membranes and nuclei are delimited respectively by the blue and red ROIs. Shortest microwire to cell membrane/nucleus distances are shown in yellow. Scale bar 20 µm (**B**) Intracellular elasticity (n=41, N=7) and (**C**) viscosity (n=39, N=7) in subconfluent monolayers, as a function of the microwire's shortest distance to cell membrane d_M. Non-significant Spearman's correlation coefficient for both sets of parameters ($P \geq 0.05$). (**D**) Intracellular elasticity (n=41, N=7) and (**E**) viscosity (n=39, N=7) in subconfluent monolayers, as a function of the microwire's shortest distance to the cell nucleus. Non-significant Spearman's correlation coefficient for intracellular elasticity ($P \geq 0.05$), significant for intracellular viscosity ($P<0.05$). (**F**) Intracellular viscosity in subconfluent monolayers (yellow, n=39, N=7) and highly dense monolayers (red, n=23, N=3). The middle distribution (shades of purple) corresponds to subconfluent values obtained for microwires located in the vicinity of the cell nucleus (below 1.5 µm). Colorbar scale indicates the microwire to nucleus distance. Datapoints shown with geometric mean and geometric standard deviation. Unpaired Student's t-test on log values. **** P< 0.0001, n.s P≥0.05.

Figure S10:

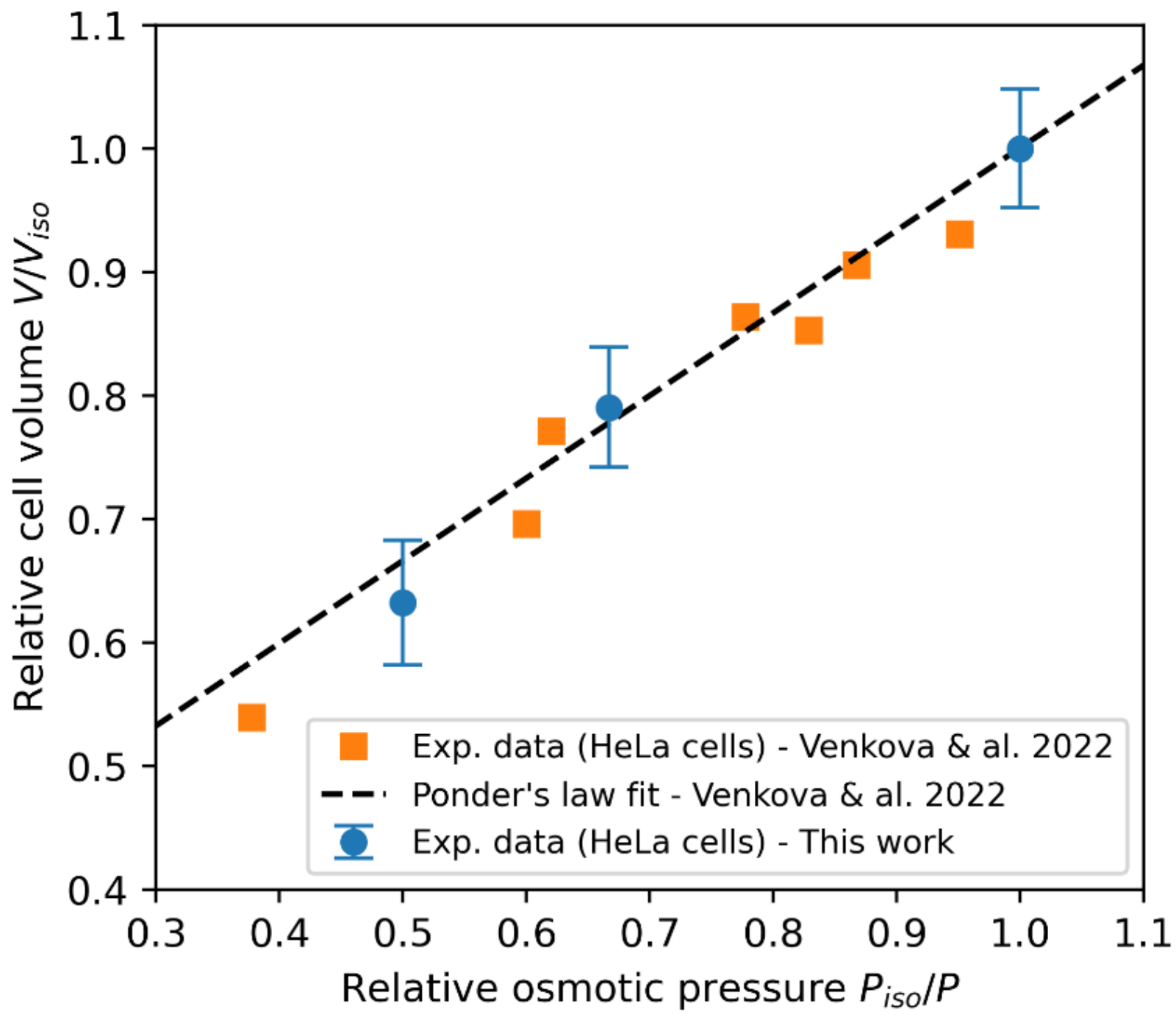

Fig. S10. Relative cell volume (HeLa) as a function of hyperosmotic pressure, measured by FXm (82) **and fluorescent z-stack thresholding**

Comparison between HeLa cell volume changes following hyperosmotic shocks using our method (blue, fluorescent z-stack thresholding) and results from Venkova et al. 2022 (*82*) (orange), which were obtained using the method of external fluorescence reduction (FXm), as developed by M. Piel. Plotted experimental points for this study (blue) correspond to the mean and standard deviation (N=4 replicate each experimental condition). The Ponder's law fit used in Venkova et al. (*82*) is represented by the dotted line.

Table S1

Reference	Cell type	Input perturbation	Storage modulus (Pa)	Loss modulus (Pa)	Dynamic viscosity (Pa.s)
**(11)**	*HeLa*	Oscillatory optical trap	**50 Pa** *(10 Hz)*	9 Pa *(100 mHz)*	**15 Pa.s**
**(14)**	*RPE-1*	Single-step displacement	**4 Pa** *(N.A)*	N.A	**30 Pa.s**
**(66)**	*A7r5*	Oscillatory optical trap	**2 Pa** *(10 Hz)*	0,7 Pa *(300 mHz)*	**0,4 Pa.s**
**(85)**	*Fibroblast*	Oscillatory optical trap	**6 Pa** *(10 Hz)*	1,5 Pa *(100 mHz)*	**2 Pa.s**
**(86)**	*Neutrophil*	Single-step displacement	**6 Pa** *(N.A)*	N.A	**0,7 Pa.s**
**(87)**	*HeLa*	Oscillatory optical trap	**60 Pa** *(10 Hz)*	7 Pa *(100 mHz)*	**11 Pa.s**

Table S1. Literature summary of intracellular mechanical properties in mammalian cells measured by optical tweezers

Summary of average values reported in the literature (*11, 14, 66, 85-87*) for the intracellular storage and loss modulus measured in mammalian cells by optical tweezers (single step displacement or oscillatory input). Whenever possible, the reported values are accompanied by the frequency at which they were measured. The estimation of dynamic viscosity corresponds to the loss modulus divided by the frequency at which it was probed.

Movie S1.

Microwires (yellow arrows) internalized in subconfluent MDCK WT, subjected to a homogeneous 12 mT magnetic field rotating at 2.7 mHz. At this frequency (i.e below the critical frequency ω_c), shown microwires are in the synchronous regime and undergo full 360° rotations by following the magnetic field alignment with a certain phase delay. 5 minutes movie length at 1 frame per 6 seconds. Scale bar, 10 µm.

Movie S2.

Microwires (yellow arrows) internalized in subconfluent MDCK WT, subjected to a homogeneous 12 mT magnetic field rotating at 84 mHz. At this frequency (i.e above the critical frequency ω_c), shown microwires are in the asynchronous regime and have an oscillatory motion as they are unable to keep up with the fast-rotating magnetic field due to cytoplasmic viscosity. 1 minute movie length at 1 frame per second. Scale bar, 10 µm.

Movie S3.

Microwires (yellow arrows) internalized in subconfluent MDCK WT, subjected to a homogeneous 12 mT magnetic field rotating at 150 mHz. Cells are here under a 1 Osm/L hypertonic shock. Internalized microwires have become essentially motionless following hypertonic shock induction. As a control, a non-internalized microwire exhibits standard oscillatory motion from sticking to the coverslip surface. 1 minute movie length at 1 frame per second. Scale bar, 10 µm.

Movie S4.

Microwires (yellow arrows) internalized in subconfluent MDCK WT, subjected to a homogeneous 12 mT magnetic field rotating at 150 mHz. Cells are in isotonic culture medium following a 1 Osm/L hypertonic shock. Internalized microwires have recovered their expected oscillatory motion at this frequency. 1 minute movie length at 1 frame per second. Scale bar, 10 µm.

Movie S5.

Example of internalized (green arrows) and non-internalized (red arrow) microwires in a subconfluent MDCK monolayer. Microwires are here subjected to a homogeneous 12 mT magnetic field rotating at 150 mHz. 1 minute movie length at 1 frame per second. Scale bar, 5 µm.

REFERENCES

1. J. Hwang, J. Kim, B. J. Sung, Dynamics of highly polydisperse colloidal suspensions as a model system for bacterial cytoplasm. *Phys. Rev. E* **94**, 022614 (2016).

2. C. Alfano, Y. Fichou, K. Huber, M. Weiss, E. Spruijt, S. Ebbinghaus, G. De Luca, M. A. Morando, V. Vetri, P. A. Temussi, A. Pastore, Molecular crowding: The history and development of a scientific paradigm. *Chem. Rev.* **124**, 3186–3219 (2024).

3. S. S. Mogre, A. I. Brown, E. F. Koslover, Getting around the cell: Physical transport in the intracellular world. *Phys. Biol.* **17**, 061003 (2020).

4. Y. Mano, Cytoplasmic regulation and cyclic variation in protein synthesis in the early cleavage stage of the sea urchin embryo. *Dev. Biol.* **22**, 433–460 (1970).

5. G. G. Fuller, J. K. Kim, Compartmentalization and metabolic regulation of glycolysis. *J. Cell Sci.* **134**, jcs258469 (2021).

6. X. Hu, J. Li, M. Fu, X. Zhao, W. Wang, The JAK/STAT signaling pathway: From bench to clinic. *Signal Transduct. Target. Ther.* **6**, 402 (2021).

7. M. A. Eskandari, J. Fischer, N. Veyret, D. Marx, T. Betz, Active intracellular mechanics—A key to cellular function and organization. *J. Cell Sci.* **138**, jcs263874 (2025).

8. M. I. Arjona, J. Najafi, N. Minc, Cytoplasm mechanics and cellular organization. *Curr. Opin. Cell Biol.* **85**, 102278 (2023).

9. J. Hu, S. Jafari, Y. Han, A. J. Grodzinsky, S. Cai, M. Guo, Size- and speed-dependent mechanical behavior in living mammalian cytoplasm. *Proc. Natl. Acad. Sci. U.S.A.* **114**, 9529–9534 (2017).

10. N. Thekkethil, J. Köry, M. Guo, P. S. Stewart, N. A. Hill, X. Luo, Modelling the rheology of living cell cytoplasm: Poroviscoelasticity and fluid-to-solid transition. *Biomech. Model. Mechanobiol.* **23**, 1551–1569 (2024).

11. H. Ebata, K. Umeda, K. Nishizawa, W. Nagao, S. Inokuchi, Y. Sugino, T. Miyamoto, D. Mizuno, Activity-dependent glassy cell mechanics I: Mechanical properties measured with active microrheology. *Biophys. J.* **122**, 1781–1793 (2023).

12. J. Najafi, S. Dmitrieff, N. Minc, Size- and position-dependent cytoplasm viscoelasticity through hydrodynamic interactions with the cell surface. *Proc. Natl. Acad. Sci. U.S.A.* **120**, e2216839120 (2023).

13. C. Verdier, Rheological properties of living materials. from cells to tissues. *Comput. Math. Methods Med.* **5**, 67–91 (2003).

14. K. Mandal, A. Asnacios, B. Goud, J.-B. Manneville, Mapping intracellular mechanics on micropatterned substrates. *Proc. Natl. Acad. Sci. U.S.A.* **113**, E7159–E7168 (2016).

15. T. Liu, X. Liu, D. R. Spring, X. Qian, J. Cui, Z. Xu, Quantitatively mapping cellular viscosity with detailed organelle information via a designed PET fluorescent probe. *Sci. Rep.* **4**, 5418 (2014).

16. I. Alshareedah, W. M. Borcherds, S. R. Cohen, A. Singh, A. E. Posey, M. Farag, A. Bremer, G. W. Strout, D. T. Tomares, R. V. Pappu, T. Mittag, P. R. Banerjee, Sequence-specific interactions determine viscoelasticity and ageing dynamics of protein condensates. *Nat. Phys.* **20**, 1482–1491 (2024).

17. P. Kollmannsberger, C. T. Mierke, B. Fabry, Nonlinear viscoelasticity of adherent cells is controlled by cytoskeletal tension. *Soft Matter* **7**, 3127–3132 (2011).

18. O. Destrian, N. Moisan, R.-M. Mège, B. Ladoux, B. Goyeau, M. Chabanon, Cytoplasmic crowding acts as a porous medium reducing macromolecule diffusion. *Proc. Natl. Acad. Sci. U.S.A.* **123**, e2519599123 (2026).

19. C. Handler, C. Testi, G. Scarcelli, Advantages of integrating Brillouin microscopy in multimodal mechanical mapping of cells and tissues. *Curr. Opin. Cell Biol.* **88**, 102341 (2024).

20. B. N. Narasimhan, M. S. Ting, T. Kollmetz, M. S. Horrocks, A. E. Chalard, J. Malmström, Mechanical characterization for cellular mechanobiology: Current trends and future prospects. *Front. Bioeng. Biotechnol.* **8**, 595978 (2020).

21. Y. Mao, P. Nielsen, J. Ali, Passive and active microrheology for biomedical systems. *Front. Bioeng. Biotechnol.* **10**, 916354 (2022).

22. T. M. Muenker, B. E. Vos, T. Betz, Intracellular mechanical fingerprint reveals cell type specific mechanical tuning. *eLife* **13**, RP97416 (2024).

23. M. Delarue, G. P. Brittingham, S. Pfeffer, I. V. Surovtsev, S. Pinglay, K. J. Kennedy, M. Schaffer, J. I. Gutierrez, D. Sang, G. Poterewicz, J. K. Chung, J. M. Plitzko, J. T. Groves, C. Jacobs-Wagner, B. D. Engel, L. J. Holt, mTORC1 controls phase separation and the biophysical properties of the cytoplasm by tuning crowding. *Cell* **174**, 338–349.e20 (2018).

24. B. E. Vos, T. M. Muenker, T. Betz, Characterizing intracellular mechanics via optical tweezers-based microrheology. *Curr. Opin. Cell Biol.* **88**, 102374 (2024).

25. A. R. Bausch, W. Möller, E. Sackmann, Measurement of local viscoelasticity and forces in living cells by magnetic tweezers. *Biophys. J.* **76**, 573–579 (1999).

26. H. Zhou, R. Liu, Y. Xu, J. Fan, X. Liu, L. Chen, Q. Wei, Viscoelastic mechanics of living cells. *Phys. Life Rev.* **53**, 91–116 (2025).

27. S. Hurst, B. E. Vos, M. Brandt, T. Betz, Intracellular softening and increased viscoelastic fluidity during division. *Nat. Phys.* **17**, 1270–1276 (2021).

28. J. Xie, J. Najafi, R. Le Borgne, J.-M. Verbavatz, C. Durieu, J. Sallé, N. Minc, Contribution of cytoplasm viscoelastic properties to mitotic spindle positioning. *Proc. Natl. Acad. Sci. U.S.A.* **119**, e2115593119 (2022).

29. A. Al Jord, G. Letort, S. Chanet, F.-C. Tsai, C. Antoniewski, A. Eichmuller, C. Da Silva, J.-R. Huynh, N. S. Gov, R. Voituriez, M.-E. Terret, M.-H. Verlhac, Cytoplasmic forces functionally reorganize nuclear condensates in oocytes. *Nat. Commun.* **13**, 5070 (2022).

30. H. Tanaka, Viscoelastic phase separation in biological cells. *Commun. Phys.* **5**, 167 (2022).

31. L. Pieuchot, J. Lai, R. A. Loh, F. Y. Leong, K.-H. Chiam, J. Stajich, G. Jedd, Cellular subcompartments through cytoplasmic streaming. *Dev. Cell* **34**, 410–420 (2015).

32. A. T. Molines, J. Lemière, M. Gazzola, I. E. Steinmark, C. H. Edrington, C.-T. Hsu, P. Real-Calderon, K. Suhling, G. Goshima, L. J. Holt, M. Thery, G. J. Brouhard, F. Chang, Physical properties of the cytoplasm modulate the rates of microtubule polymerization and depolymerization. *Dev. Cell* **57**, 466–479.e6 (2022).

33. M. Dessard, J.-B. Manneville, J.-F. Berret, Cytoplasmic viscosity is a potential biomarker for metastatic breast cancer cells. *Nanoscale Adv.* **6**, 1727–1738 (2024).

34. L. Petitjean, M. Reffay, E. Grasland-Mongrain, M. Poujade, B. Ladoux, A. Buguin, P. Silberzan, Velocity fields in a collectively migrating epithelium. *Biophys. J.* **98**, 1790–1800 (2010).

35. T. E. Angelini, E. Hannezo, X. Trepat, M. Marquez, J. J. Fredberg, D. A. Weitz, Glass-like dynamics of collective cell migration. *Proc. Natl. Acad. Sci. U.S.A.* **108**, 4714–4719 (2011).

36. J.-A. Park, J. H. Kim, D. Bi, J. A. Mitchel, N. T. Qazvini, K. Tantisira, C. Y. Park, M. McGill, S.-H. Kim, B. Gweon, J. Notbohm, R. Steward Jr, S. Burger, S. H. Randell, A. T. Kho, D. T. Tambe, C. Hardin, S. A. Shore, E. Israel, D. A. Weitz, D. J. Tschumperlin, E. P. Henske, S. T. Weiss, M. L. Manning, J. P. Butler, J. M. Drazen, J. J. Fredberg, Unjamming and cell shape in the asthmatic airway epithelium. *Nat. Mater.* **14**, 1040–1048 (2015).

37. O. Du Roure, A. Saez, A. Buguin, R. H. Austin, P. Chavrier, P. Siberzan, B. Ladoux, Force mapping in epithelial cell migration. *Proc. Natl. Acad. Sci. U.S.A.* **102**, 2390–2395 (2005).

38. X. Trepat, M. R. Wasserman, T. E. Angelini, E. Millet, D. A. Weitz, J. P. Butler, J. J. Fredberg, Physical forces during collective cell migration. *Nat. Phys.* **5**, 426–430 (2009).

39. S. Nehls, H. Nöding, S. Karsch, F. Ries, A. Janshoff, Stiffness of MDCK II cells depends on confluency and cell size. *Biophys. J.* **116**, 2204–2211 (2019).

40. S. J. Chisolm, E. Guo, V. Subramaniam, K. D. Schulze, T. E. Angelini, Elastic modulus versus cell packing density in MDCK epithelial monolayers. *J. Biomech.* **184**, 112648 (2025).

41. E. Rodriguez-Boulan, I. G. Macara, Organization and execution of the epithelial polarity programme. *Nat. Rev. Mol. Cell Biol.* **15**, 225–242 (2014).

42. Y. Fan, T. Meyer, Molecular control of cell density-mediated exit to quiescence. *Cell Rep.* **36**, 109436 (2021).

43. J. Devany, M. J. Falk, L. J. Holt, A. Murugan, M. L. Gardel, Epithelial tissue confinement inhibits cell growth and leads to volume-reducing divisions. *Dev. Cell* **58**, 1462–1476.e8 (2023).

44. R. M. Sarate, J. Hochstetter, M. Valet, A. Hallou, Y. Song, N. Bansaccal, M. Ligare, M. Aragona, D. Engelman, A. Bauduin, O. Campàs, B. D. Simons, C. Blanpain, Dynamic regulation of tissue fluidity controls skin repair during wound healing. *Cell* **187**, 5298–5315.e19 (2024).

45. S. E. Lim, P. Vicente-Munuera, Y. Mao, Forced back into shape: Mechanics of epithelial wound repair. *Curr. Opin. Cell Biol.* **87**, 102324 (2024).

46. Y. Fujii, Y. Ochi, M. Tuchiya, M. Kajita, Y. Fujita, Y. Ishimoto, T. Okajima, Spontaneous spatial correlation of elastic modulus in jammed epithelial monolayers observed by AFM. *Biophys. J.* **116**, 1152–1158 (2019).

47. J.-F. Berret, Local viscoelasticity of living cells measured by rotational magnetic spectroscopy. *Nat. Commun.* **7**, 10134 (2016).

48. L. Chevry, R. Colin, B. Abou, J.-F. Berret, Intracellular micro-rheology probed by micron-sized wires. *Biomaterials* **34**, 6299–6305 (2013).

49. S. Goswami, S. Mukherjee, N. Ahmed, M. Sreepadmanabh, T. Bhattacharjee, R. Bhat, D. Saini, A. Ghosh, Frequency-chirped actuation of chiral magnetic microbots for viscosity mapping in heterogenous media: From model fluids to living cells. *ACS Nano* **19**, 36373–36383 (2025).

50. M. Poujade, E. Grasland-Mongrain, A. Hertzog, J. Jouanneau, P. Chavrier, B. Ladoux, A. Buguin, P. Silberzan, Collective migration of an epithelial monolayer in response to a model wound. *Proc. Natl. Acad. Sci. U.S.A.* **104**, 15988–15993 (2007).

51. D. L. Nikolić, A. N. Boettiger, D. Bar-Sagi, J. D. Carbeck, S. Y. Shvartsman, Role of boundary conditions in an experimental model of epithelial wound healing. *Am. J. Physiol. Cell Physiol.* **291**, C68–C75 (2006).

52. L. Chevry, N. K. Sampathkumar, A. Cebers, J.-F. Berret, Magnetic wire-based sensors for the microrheology of complex fluids. *Phys. Rev. E Stat. Nonlin. Soft Matter Phys.* **88**, 062306 (2013).

53. A. Puliafito, L. Hufnagel, P. Neveu, S. Streichan, A. Sigal, D. K. Fygenson, B. I. Shraiman, Collective and single cell behavior in epithelial contact inhibition. *Proc. Natl. Acad. Sci. U.S.A.* **109**, 739–744 (2012).

54. D. A. Fletcher, R. D. Mullins, Cell mechanics and the cytoskeleton. *Nature* **463**, 485–492 (2010).

55. E. A. Katrukha, M. Mikhaylova, H. X. van Brakel, P. M. van Bergen En Henegouwen, A. Akhmanova, C. C. Hoogenraad, L. C. Kapitein, Probing cytoskeletal modulation of passive and active intracellular dynamics using nanobody-functionalized quantum dots. *Nat. Commun.* **8**, 14772 (2017).

56. B. L. Doss, M. Pan, M. Gupta, G. Grenci, R.-M. Mège, C. T. Lim, M. P. Sheetz, R. Voituriez, B. Ladoux, Cell response to substrate rigidity is regulated by active and passive cytoskeletal stress. *Proc. Natl. Acad. Sci. U.S.A.* **117**, 12817–12825 (2020).

57. B. R. Brückner, H. Nöding, A. Janshoff, Viscoelastic properties of confluent MDCK II cells obtained from force cycle experiments. *Biophys. J.* **112**, 724–735 (2017).

58. C. Rotsch, M. Radmacher, Drug-induced changes of cytoskeletal structure and mechanics in fibroblasts: An atomic force microscopy study. *Biophys. J.* **78**, 520–535 (2000).

59. A. Pietuch, B. R. Brückner, T. Fine, I. Mey, A. Janshoff, Elastic properties of cells in the context of confluent cell monolayers: Impact of tension and surface area regulation. *Soft Matter* **9**, 11490 (2013).

60. U. S. Potard, J. P. Butler, N. Wang, Cytoskeletal mechanics in confluent epithelial cells probed through integrins and E-cadherins. *Am. J. Physiol.* **272**, C1654–C1663 (1997).

61. Y. Xie, T. Shu, T. Liu, M.-C. Spindler, J. Mahamid, G. M. Hocky, D. Gresham, L. J. Holt, Polysome collapse and RNA condensation fluidize the cytoplasm. *Mol. Cell* **84**, 2698–2716.e9 (2024).

62. L. Balasubramaniam, S. Monfared, A. Ardaševa, C. Rosse, A. Schoenit, T. Dang, C. Maric, M. Hautefeuille, L. Kocgozlu, R. Chilupuri, S. Dubey, E. Marangoni, B. L. Doss, P. Chavrier, R.-M. Mége, A. Doostmohammadi, B. Ladoux, Dynamic forces shape the survival fate of eliminated cells. *Nat. Phys.* **21**, 269–278 (2025).

63. H. Wu, C. Duclut, G. Arkowitz, R. Chilupuri, T. Dang, J. Prost, B. Ladoux, R.-M. Mège, Regulation of epithelial tissue homeostasis by active transepithelial transport. *Proc. Natl. Acad. Sci. U.S.A.* **122**, e2503156122 (2025).

64. T. G. Mason, D. A. Weitz, Linear viscoelasticity of colloidal hard sphere suspensions near the glass transition. *Phys. Rev. Lett.* **75**, 2770–2773 (1995).

65. P. Cassagnau, Linear viscoelasticity and dynamics of suspensions and molten polymers filled with nanoparticles of different aspect ratios. *Polymer* **54**, 4762–4775 (2013).

66. M. Guo, A. F. Pegoraro, A. Mao, E. H. Zhou, P. R. Arany, Y. Han, D. T. Burnette, M. H. Jensen, K. E. Kasza, J. R. Moore, F. C. Mackintosh, J. J. Fredberg, D. J. Mooney, J. Lippincott-Schwartz, D. A. Weitz, Cell volume change through water efflux impacts cell stiffness and stem cell fate. *Proc. Natl. Acad. Sci. U.S.A.* **114**, E8618–E8627 (2017).

67. E. Ponder, G. Saslow, The measurement of red cell volume. *J. Physiol.* **70**, 18–37 (1930).

68. C. Roffay, G. Molinard, K. Kim, M. Urbanska, V. Andrade, V. Barbarasa, P. Nowak, V. Mercier, J. García-Calvo, S. Matile, R. Loewith, A. Echard, J. Guck, M. Lenz, A. Roux, Passive coupling of membrane tension and cell volume during active response of cells to osmosis. *Proc. Natl. Acad. Sci. U.S.A.* **118**, e2103228118 (2021).

69. P.-H. Wu, D. R.-B. Aroush, A. Asnacios, W.-C. Chen, M. E. Dokukin, B. L. Doss, P. Durand-Smet, A. Ekpenyong, J. Guck, N. V. Guz, P. A. Janmey, J. S. H. Lee, N. M. Moore, A. Ott, Y.-C. Poh, R. Ros, M. Sander, I. Sokolov, J. R. Staunton, N. Wang, G. Whyte, D. Wirtz, A comparison of methods to assess cell mechanical properties. *Nat. Methods* **15**, 491–498 (2018).

70. S. Mathieu, J.-B. Manneville, Intracellular mechanics: Connecting rheology and mechanotransduction. *Curr. Opin. Cell Biol.* **56**, 34–44 (2019).

71. W.-J. Lin, H. Yu, A. Pathak, Gradients in cell density and shape transitions drive collective cell migration into confining environments. *Soft Matter* **21**, 719–728 (2025).

72. L. J. Holt, M. Delarue, Macromolecular crowding: Sensing without a sensor. *Curr. Opin. Cell Biol.* **85**, 102269 (2023).

73. K. Nishizawa, K. Fujiwara, M. Ikenaga, N. Nakajo, M. Yanagisawa, D. Mizuno, Universal glass-forming behavior of in vitro and living cytoplasm. *Sci. Rep.* **7**, 15143 (2017).

74. L. Atia, J. J. Fredberg, N. S. Gov, A. F. Pegoraro, Are cell jamming and unjamming essential in tissue development? *Cells Dev.* **168**, 203727 (2021).

75. M. Zhu, H. Tao, M. Samani, M. Luo, X. Wang, S. Hopyan, Y. Sun, Spatial mapping of tissue properties in vivo reveals a 3D stiffness gradient in the mouse limb bud. *Proc. Natl. Acad. Sci. U.S.A.* **117**, 4781–4791 (2020).

76. E. H. Barriga, K. Franze, G. Charras, R. Mayor, Tissue stiffening coordinates morphogenesis by triggering collective cell migration in vivo. *Nature* **554**, 523–527 (2018).

77. N. Srivastava, L. Calabrese, C. N. Plancke, R. Rollin, L. Venkova, K. Havas, M. Cosentino Lagomarsino, M. Piel, A dual homeostatic regulation of dry mass and volume defines a target

density in proliferating mammalian cells. bioRxiv 2025.04.24.650395 [Preprint] (2025). https://doi.org/10.1101/2025.04.24.650395.

78. A. Mukherjee, Y. Huang, J. Elgeti, S. Oh, J. G. Abreu, L. Ammar, A. R. Neliat, J. Schüttler, D.-D. Su, C. Dupre, N. C. Benites, X. Liu, L. Peshkin, M. Barboiu, H. Stocker, M. W. Kirschner, M. Basan, Membrane potential mediates the cellular response to mechanical pressure. *Cell* **189**, 143–160.e22 (2026).

79. W. Eimer, R. Pecora, Rotational and translational diffusion of short rodlike molecules in solution: Oligonucleotides. *J. Chem. Phys.* **94**, 2324–2329 (1991).

80. P. Berto, H. Rigneault, M. Guillon, Wavefront sensing with a thin diffuser. *Opt. Lett.* **42**, 5117–5120 (2017).

81. T. Wu, M. Guillon, C. Gentner, H. Rigneault, G. Tessier, P. Bon, P. Berto, 3D nanoparticle superlocalization with a thin diffuser. *Opt. Lett.* **47**, 3079–3082 (2022).

82. L. Venkova, A. S. Vishen, S. Lembo, N. Srivastava, B. Duchamp, A. Ruppel, A. Williart, S. Vassilopoulos, A. Deslys, J. M. Garcia Arcos, A. Diz-Muñoz, M. Balland, J.-F. Joanny, D. Cuvelier, P. Sens, M. Piel, A mechano-osmotic feedback couples cell volume to the rate of cell deformation. *eLife* **11**, e72381 (2022).

83. L. Shen, J. R. Turner, Actin depolymerization disrupts tight junctions via caveolae-mediated endocytosis. *Mol. Biol. Cell* **16**, 3919–3936 (2005).

84. C. Saunders, L. E. Limbird, Disruption of microtubules reveals two independent apical targeting mechanisms for G-protein-coupled receptors in polarized renal epithelial cells. *J. Biol. Chem.* **272**, 19035–19045 (1997).

85. L. Chaubet, A. R. Chaudhary, H. K. Heris, A. J. Ehrlicher, A. G. Hendricks, Dynamic actin cross-linking governs the cytoplasm’s transition to fluid-like behavior. *Mol. Biol. Cell* **31**, 1744–1752 (2020).

86. M. Yanai, J. P. Butler, T. Suzuki, H. Sasaki, H. Higuchi, Regional rheological differences in locomoting neutrophils. *Am. J. Physiol. Cell Physiol.* **287**, C603–C611 (2004).

87. H. Ebata, K. Nishizawa, F. A. S. Van Esterik, T. Maeda, Y. Tao, S. Inokuchi, H. Ise, D. Mizuno, Single power-law rheology of crowded cytoplasm in living cells. *Cell Rep. Phys. Sci.* **7**, 103059 (2026).
